# Blunted medial prefrontal cortico-limbic reward-related effective connectivity and depression

**DOI:** 10.1093/brain/awaa106

**Published:** 2020-05-08

**Authors:** Samuel Rupprechter, Liana Romaniuk, Peggy Series, Yoriko Hirose, Emma Hawkins, Anca-Larisa Sandu, Gordon D Waiter, Christopher J McNeil, Xueyi Shen, Mathew A Harris, Archie Campbell, David Porteous, Jennifer A Macfarlane, Stephen M Lawrie, Alison D Murray, Mauricio R Delgado, Andrew M McIntosh, Heather C Whalley, J Douglas Steele

**Affiliations:** a1 School of Informatics, University of Edinburgh, Edinburgh, UK; a2 Division of Psychiatry, University of Edinburgh, Edinburgh, UK; a3 Biomedical Imaging Centre, University of Aberdeen, Aberdeen, UK; a4 Centre for Genomic and Experimental Medicine, University of Edinburgh, Edinburgh, UK; a5 Division of Imaging Science and Technology, Medical School, University of Dundee, Dundee, UK; a6 Department of Psychology, Rutgers University, Piscataway, NJ, USA

**Keywords:** RDoC, effective connectivity, reinforcement learning, MDD, DCM

## Abstract

Major depressive disorder is a leading cause of disability and significant mortality, yet mechanistic understanding remains limited. Over the past decade evidence has accumulated from case-control studies that depressive illness is associated with blunted reward activation in the basal ganglia and other regions such as the medial prefrontal cortex. However it is unclear whether this finding can be replicated in a large number of subjects. The functional anatomy of the medial prefrontal cortex and basal ganglia has been extensively studied and the former has excitatory glutamatergic projections to the latter. Reduced effect of glutamatergic projections from the prefrontal cortex to the nucleus accumbens has been argued to underlie motivational disorders such as depression, and many prominent theories of major depressive disorder propose a role for abnormal cortico-limbic connectivity. However, it is unclear whether there is abnormal reward-linked effective connectivity between the medial prefrontal cortex and basal ganglia related to depression. While resting state connectivity abnormalities have been frequently reported in depression, it has not been possible to directly link these findings to reward-learning studies. Here, we tested two main hypotheses. First, mood symptoms are associated with blunted striatal reward prediction error signals in a large community-based sample of recovered and currently ill patients, similar to reports from a number of studies. Second, event-related directed medial prefrontal cortex to basal ganglia effective connectivity is abnormally increased or decreased related to the severity of mood symptoms. Using a Research Domain Criteria approach, data were acquired from a large community-based sample of subjects who participated in a probabilistic reward learning task during event-related functional MRI. Computational modelling of behaviour, model-free and model-based functional MRI, and effective connectivity dynamic causal modelling analyses were used to test hypotheses. Increased depressive symptom severity was related to decreased reward signals in areas which included the nucleus accumbens in 475 participants. Decreased reward-related effective connectivity from the medial prefrontal cortex to striatum was associated with increased depressive symptom severity in 165 participants. Decreased striatal activity may have been due to decreased cortical to striatal connectivity consistent with glutamatergic and cortical-limbic related theories of depression and resulted in reduced direct pathway basal ganglia output. Further study of basal ganglia pathophysiology is required to better understand these abnormalities in patients with depressive symptoms and syndromes.

## Teaser

Clinical studies have reported altered reward processing in major depressive disorder. Using dynamic causal modelling, Rupprecher *et al.* show that blunted reward-related effective connectivity from the medial prefrontal cortex to the striatum is associated with increased severity of depressive symptoms.

## Introduction

Major depressive disorder (MDD) is a leading cause of disability worldwide and a cause of significant mortality, yet there is wide agreement that its treatment has not changed fundamentally in over half a century (Steele and Paulus, 2019). However, there is now substantial evidence from a series of independent neuroimaging studies acquired over more than a decade, that MDD is associated with blunted reward signals in the medial prefrontal cortex (mPFC) and particularly in the basal ganglia (Forbes *et al.*, 2006; Steele *et al.*, 2007; Kumar *et al.*, 2008, 2018; Pizzagalli *et al.*, 2009; Eshel and Roiser, 2010; Gradin *et al.*, 2011; Zhang *et al.*, 2013; Pizzagalli, 2014; Johnston *et al.*, 2015; Stringaris *et al.*, 2015; Rothkirch *et al.*, 2017; Keren *et al.*, 2018), consistent with earlier large behavioural decision-making studies (Forbes *et al.*, 2007) and the conclusions of a large behavioural meta-analysis on decision making in depression (Huys *et al.*, 2013).

In addition to studies on task-based reward processing, there are now many studies of resting state connectivity in MDD (Kaiser *et al.*, 2015, 2016); however, the link between blunted reward signals in task-based reward learning studies and possible event-related connectivity abnormalities in MDD remains unclear. Functional connectivity abnormalities present during a resting state study may be different from event-related effective connectivity abnormalities during reinforcement learning studies involving valenced (reward or punishment) feedback. Recognition of different types of connectivity is important, because a number of prominent theories propose a role for abnormal connectivity in depression (Mayberg *et al.*, 2005; Disner *et al.*, 2011; Roiser *et al.*, 2012; Russo and Nestler, 2013; Pizzagalli, 2014) without distinguishing different types of connectivity. While most MDD neuroimaging studies have focused on resting state undirected functional connectivity, a recent exception reported blunted striatal reward prediction error signals and blunted reward-linked ventral tegmental area to striatal event-related connectivity (Kumar *et al.*, 2018). The ventral tegmental area projection is dopaminergic, has been extensively studied in animals and is part of the classical basal ganglia thalamocortical circuit (Alexander and Crutcher, 1990; Alexander *et al.*, 1990).

Event-related functional MRI studies of reward learning tasks in humans report consistent activation of the basal ganglia and rostral-subgenual mPFC (Kim *et al.*, 2011; Johnston *et al.*, 2015), which are prominent parts of the limbic basal ganglia thalamocortical circuits. The mPFC to basal ganglia projection has been studied in animals and is glutamatergic (Alexander and Crutcher, 1990; Alexander *et al.*, 1990). We were particularly interested in whether the effective connectivity for this medial prefrontal projection was abnormal in volunteers with increased depressive symptoms and decreased brain reward responses. The rostral cingulate is important as influential PET imaging studies reported abnormal metabolic activity in MDD (Drevets *et al.*, 1997; Mayberg *et al.*, 2005), which motivated a subgenual deep brain stimulation international treatment trial (Holtzheimer *et al.*, 2017).

Here we analysed behaviour and functional MRI data from a large community-based sample of volunteers. A dimensional approach was chosen because the Research Domain Criteria (RDoC) (Insel *et al.*, 2010) approach aims to explore the ‘full range of variation from normal to abnormal’, recognizing current diagnostic systems ‘do not adequately reflect relevant neurobiological and behavioural systems—impeding not only research on aetiology and pathophysiology but also the development of new treatments’ (Cuthbert and Insel, 2013). Our behavioural task included an aspect of control, as there have been reports that reward processing may be affected by whether an individual values making their own choices and in our previous work (Romaniuk *et al.*, 2019), we found evidence for an association between the inherent value of choice and activation in MDD-related regions including striatum and mPFC.

Two primary hypotheses were tested: (i) mood symptoms are associated with blunted reward and/or reward prediction error (RPE) signals, similar to reports from a number of clinical studies; and (ii) there is abnormal (increased or decreased) event-related, directed rostral anterior cingulate to basal ganglia effective connectivity, linked to the severity of mood symptoms. In addition motivated by our previous work (Romaniuk *et al.*, 2019), we also tested the hypothesis that (iii) individuals learned differently from outcomes depending on whether they had control over decisions, related to the presence of mood symptoms.

## Materials and methods

### Participants

Subjects were recruited via the Stratifying Resilience and Depression Longitudinally (STRADL) study (Navrady *et al.*, 2018). The STRADL clinical cohort is a subset of the Generation Scotland Scottish Family Health Study who were originally recruited in Scotland from 2006 to 2011, aged over 18 at the time (Smith *et al.*, 2013). Generation Scotland participants residing in north east Scotland (Grampian and Tayside areas) were invited to attend a clinic in Aberdeen or Dundee for MRI scanning, other testing and sample collection.

### Clinical interview and questionnaire data

All participants were assessed for a lifetime history of MDD using the Structured Clinical Interview for DSM-IV disorders (SCID) (First *et al.*, 2002). Diagnostic criteria were based on the Diagnostic and Statistical Manual of Mental Disorders (DSM-IV-TR). Participants also completed a series of questionnaires that included the Quick Inventory of Depressive Symptomatology (QIDS) (Rush *et al.*, 2003), which is 16-item inventory designed to assess the severity of depression symptoms, and the Hospital Anxiety and Depression Scale (HADS) (Zigmond and Snaith, 1983) anxiety subscale (seven items), which was used to assess symptoms of anxiety (Habota *et al.*, 2019).

### Participant selection and analyses

Computational modelling of behaviour and event-related functional MRI analyses were performed on 475 participants, which included 20 subjects with a current major depressive episode (MDE) (Table 1). For dynamic causal modelling (DCM) 165 subjects were selected who had sufficiently strong functional MRI signals in the regions of interest. Sufficiently strong signals are required for DCM to be valid, despite depressive symptoms being associated with blunting of signal strength in clinical studies. Data selection is summarized in Supplementary Fig. 9 and further described in the Supplementary material, which contains additional analyses showing that varying the inclusion criteria did not significantly influence the DCM results. Importantly, included and excluded subjects did not differ significantly with respect to QIDS depression severity scores.

**Table 1:** Demographic and clinical details

	Healthy participants	Past MDD	Current MDE
Number of subjects	345	110	20
Age, range (mean ± SD)	28–78 (60 ± 8.8)	27–72 (57 ± 8.4)	37–65 (56 ± 8.7)
Sex, female / male	177 / 168	78 / 32	16 / 4
QIDS-SR, range (mean ± SD)	0–12 (3.39 ± 2.08)	1–22 (5.41 ± 3.84)	9–21 (14.55 ± 3.79)
HADS-A, range (mean ± SD)	0–12 (3.13 ± 2.44)	0–17 (5.04 ± 3.35)	6–20 (10.65 ± 3.62)

GHQ = General Health Questionnaire; HADS = Hospital Anxiety and Depression Scale; MDE = major depressive episode; SD = standard deviation.

### Scanning and behavioural paradigms

**Figure 1:**
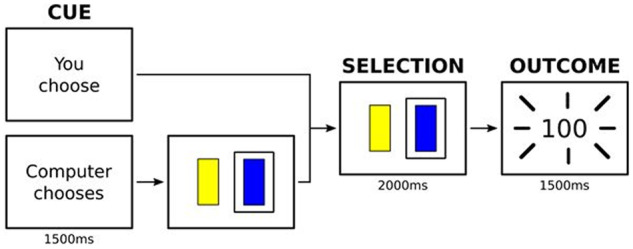
**Probabilistic reward learning task.** Subjects completed trials of a probabilistic reward learning task which involved choosing one of two stimuli. During the first phase of each trial a cue indicated whether participants would be allowed to freely make a choice between the two squares or whether the computer would choose for them and they had to follow that choice. During the second phase a choice was made or confirmed. During the third phase an outcome (‘no reward’ or ‘reward’) was presented.

T_1_-weighted images and functional MRI data were acquired at Dundee and Aberdeen Universities. For functional MRI acquisition in Dundee, a 3 T Siemens PRISMA was used with repetition time 1.56 s, echo time 22 ms, flip angle 70°, field of view 217 mm, matrix 64 × 64, 32 axial slices; in Aberdeen a 3 T Philips ACHIEVA was used with repetition time 1.56 s, echo time 26 ms, flip angle 70°, field of view 217 mm, matrix 64 × 64, 32 axial slices. Subjects completed 66 trials of a probabilistic reward learning task (Romaniuk *et al.*, 2019), which involved choosing one of two stimuli (yellow or blue squares). Participants were not told that the stimuli were associated with different reward probabilities (80% for the yellow square, 20% for the blue square) and feedback on their choices was provided by display of a number of points: 100 points for a ‘win’ or reward, 0 points for ‘no win’ or no reward. During the first phase of each trial a cue indicated whether participants would be allowed to freely make a choice between the two squares or whether the computer would choose for them and they had to follow that choice. Phases were jittered, allowing for disambiguation. The number of trials was split into 33 ‘choice’ and 33 ‘no choice’ trials with the task being summarized in Fig. 1.

### Computational modelling of behaviour

Five reinforcement learning models represented distinct hypotheses about how subjects learned during the task. The aims of the modelling were to (i) correlate model parameter estimates with depressive symptom severity scores; (ii) estimate RPE signals for use in model-based functional MRI analyses; and (iii) compare learning during choice versus no-choice trials. Model 1 assumed participants only learned from choice outcomes and ignored no-choice outcomes, model 2 assumed participants learned equally well during both choice and no-choice trials, model 3 assumed participants learned at different rates on choice versus no-choice trials, model 4 assumed reward outcomes were experienced differently depending on the choice versus no-choice condition (i.e. different ‘reward sensitivity’ parameters) and model 5 assumed both learning and outcomes were experienced differently (different learning rates and reward sensitivity parameters). Fitted parameters were maximum *a posteriori* estimates and models were compared using the integrated Bayesian Information Criterion (Huys *et al.*, 2013) (Supplementary material).

### Image preprocessing and general linear model voxel-based functional MRI analyses

SPM12 was used for analyses with functional images realigned to the first image, unwarped and slice time corrected. The T_1_-weighted structural image was segmented and functional images were co-registered to the bias corrected T_1_ image. Images were spatially normalized and smoothed using an 8-mm Gaussian kernel. Additional details are provided in the Supplementary material.

An event-related design was used for the first-level analysis. A first level general linear model (GLM) design matrix included two columns for onsets of choice or no-choice cues, four columns of possible outcomes (reward/no-reward during choice/no-choice trials), two columns for responses (button-press) during choice/no-choice trials, and one column for nuisance regressors (response time out or incorrect response during no-choice trials). Six rigid body motion realignment parameters estimated during preprocessing were included as covariates of no interest. For model-based functional MRI analyses, the four outcome columns were replaced by a single column of all outcome events and a column of the parametric modulator: reward/no-reward outcome coded as 1 or 0, or the estimated RPE signal. Events were modelled as truncated delta-functions and convolved with the SPM12 canonical haemodynamic response function without time or dispersion derivatives.

Contrast estimates from each subject’s first level analysis were taken to the second level. Of interest was (i) the reward activations and RPE encoding signals across all participants calculated using contrasts for the corresponding parametric modulator [as expected a contrast of ‘reward(choice + no-choice) outcome > no-reward(choice + no-choice) outcome’ in the GLM matrix not using parametric modulators gave similar results]; (ii) reward response during choice conditions compared to reward response during no-choice conditions [reward(choice) outcome > reward(no-choice) outcome]; and (iii) correlations with depressive symptom scores across participants.

Multiple comparisons of effects linked to depressive symptom severity were corrected using a whole brain cluster corrected threshold of *P < *0.001, comprising a simultaneous requirement for a *P < *0.05 voxel threshold and >131 contiguous supra-threshold voxels, this being estimated using Monte Carlo simulations (Supplementary material) (Slotnick *et al.*, 2003).

### Dynamic causal modelling of event-related effective connectivity

DCM (Friston *et al.*, 2007) was used to investigate how the severity of depressive symptoms was associated with a small network of three brain regions active during the task. Our connectivity hypotheses concerned between-subject level inferences, meaning we tested for an association between QIDS scores and the general task-independent connectivity (DCM ‘A’ matrix).

**Figure 2:**
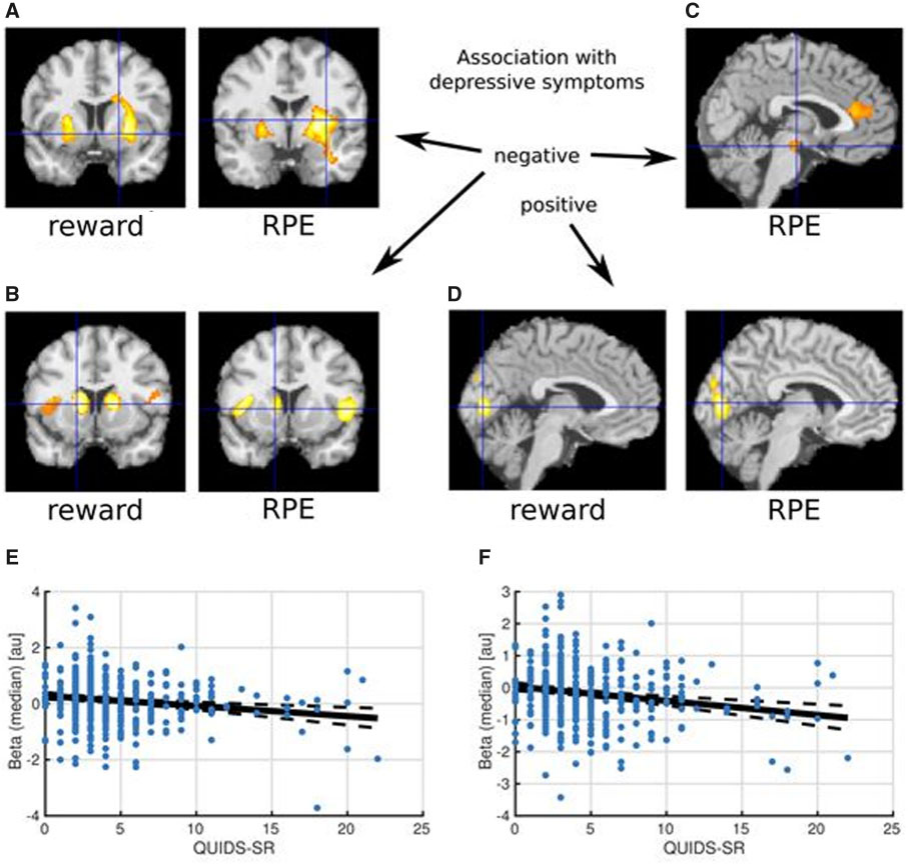
**Correlations between depressive symptom scores and reward signal encoding.** Higher depressive symptoms were associated with lower striatal reward response. (**A**) Decreased reward activation/RPE encoding signal in putamen/ventral striatum; (**B**) increased deactivation/negative RPE encoding signal in caudate and insula; (**C**) decreased RPE encoding signal in midbrain; and (**D**) increased reward activation/RPE encoding in occipital lobe, with regions significant at *P < *0.001 whole-brain corrected. (**E**) Negative correlation of QIDS scores with striatal activity (26, 4, 0) (Spearman’s *P = *−0.16, *P < *0.001). (**F**) Negative correlation of QIDS scores with striatal activity (−16, 10, 6) (Spearman’s *P = *−0.20, *P < *0.001).

Brain regions were selected to test the hypotheses of a mood linked change in effective connectivity between regions involved in the brain’s reward network. The left ventral striatum centred at MNI (−12, 10, −14) (see ‘Results’ section and Supplementary Table 2) was selected because there is extensive evidence for blunted activation in MDD (Steele *et al.*, 2007; Kumar *et al.*, 2008, 2018; Pizzagalli *et al.*, 2009; Eshel and Roiser, 2010; Gradin *et al.*, 2011; Zhang *et al.*, 2013; Pizzagalli, 2014; Johnston *et al.*, 2015; Rothkirch *et al.*, 2017; Keren *et al.*, 2018). An mPFC region was selected centred at (−2, 52, 18) (Fig. 2C and Supplementary Table 4) because this region usually coactivates with the ventral striatum on reward delivery (O’Doherty, 2004; Gradin *et al.*, 2014; Johnston *et al.*, 2015) and the mPFC has direct projections to the striatum (Alexander and Crutcher, 1990; Alexander *et al.*, 1990). In addition, a visual cortex region centred at (−8, −88, −4) (Supplementary Table 2) was chosen as the brain region receiving experimentally controlled inputs. This visual region and ventral striatum were also constrained anatomically using the pericalcarine and accumbens Freesurfer masks (Reuter *et al.*, 2012). For each participant we extracted the first principal component of the time series of 12-mm spheres, which were centred at the above MNI coordinates, but importantly were further constrained by liberal individual activation thresholds as well as the above-mentioned anatomical masks, meaning signals were only extracted from a subset of the voxels contained in the spherical regions of interest.

A bilinear DCM with one state per region and no stochastic effects was assumed and a fully connected model of nine connections including inhibitory self-connections was fitted. There are known direct excitatory glutamatergic projections from the anterior cingulate to the striatum (Alexander and Crutcher, 1990; Alexander *et al.*, 1990) and the possible effects of depression symptoms on this direct top-down connection were of particular interest. All other connections were assumed to be indirect. Four outcome types [‘reward’ (choice + no-choice trials), ‘no-reward’ (choice + no-choice), ‘choice’ (reward + no-reward), ‘no-choice’ (reward + no-reward)] were used as driving inputs to the visual cortex (‘outcome display’) (see Supplementary material for control analyses using an alternative input specification). It was assumed that each of these four outcome conditions could also modulate each of the intrinsic (endogenous, task-invariant) connections. The display of choice/no-choice cues served as additional inputs to the visual area and responses (choice/no-choice condition button presses) drove activity in any region. Inputs were mean-centred so that parameters of the endogenous (‘A’ matrix) connectivity specified the average effective connectivity between regions and the modulations (‘B’ matrix) added or subtracted from this average.

For each participant, the full DCM was fitted to the data and the percentage of the variance explained was calculated. As recommended in the SPM documentation and online SPM discussion groups (Zeidman, 2019), we only included participants for which the variance explained by the model was at least 10% (Supplementary material). The parametric empirical Bayes (PEB) framework (Friston *et al.*, 2016) was used to model commonalities and differences across participants. The group-level between-subject PEB design matrix included a column of ones, corresponding to the mean connectivity across participants, and a zero-mean centred column of our covariate of interest (QIDS depression scores). Five additional mean-centred covariates included HADS anxiety scores, age, sex, collection site and current MDE diagnosis (see Supplementary material for additional analyses without these covariates). The group-level within-subject design matrix was defined as the identity matrix, which means we assumed the covariates could potentially have an effect on every within-subject DCM parameter. The full PEB model was inverted to obtain parameter estimates and the model’s ‘free energy’.

**Figure 3:**
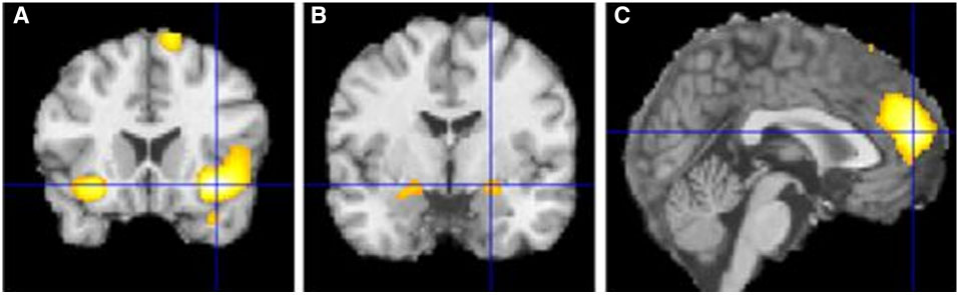
**Activations comparing choice with no-choice conditions.** Activated regions during reward outcomes during choice compared to reward outcomes during no-choice conditions: (**A**) insula, (**B**) amygdala, (**C**) mPFC. Regions significant at *P < *0.001 whole-brain corrected.

Bayesian model reduction (Friston *et al.*, 2016) was used to rapidly estimate different reduced PEB models within which certain parameters were ‘switched off’. An automatic ‘greedy’ search procedure was used to iteratively prune parameters that did not contribute to the free energy. The models identified at the final iteration were combined using Bayesian model averaging (Fig. 3A) (Penny *et al.*, 2006). Our main analysis focused on a PEB model including nine DCM (‘A’ matrix) parameters (see Supplementary material for additional analyses).

To increase confidence in our results, a large number of control analyses were performed (Supplementary material). Most notably, during these analyses different variance-explained thresholds were used, and different covariates were included in the second level design matrix (e.g. only QIDS was included as covariate). Additional control analyses also included an analysis of individual symptoms (as opposed to the QIDS sum of individual symptom scores) to address both a skew in overall QIDS scores and the possibility of correlation effects being influenced by a group-level (i.e. never-depressed healthy participants versus MDE subjects) effect. This is described in detail in the Supplementary material. Bootstrap split-sample replication was used to test the effective connectivity hypothesis.

### Data availability

The data that support the findings of this study are available by application to the Generation Scotland Access Committee (access@generationscotland.org) and Edinburgh Data Vault (https://doi.org/10.7488/8f68f1ae-0329-4b73-b189-c7288ea844d7). For further information, see Habota *et al.* (2019).

## Results

### Behavioural analyses

There was no significant Spearman’s correlation between QIDS depression score and number of rewards gained [Spearman’s ρ(475) = 0.064, *P *=* *0.164] on the task, or between QIDS score and number of missed trials [Spearman’s ρ(475) = 0.064, *P *=* *0.164], facilitating interpretation of the imaging results. Formal model comparison identified model 3, which assumed subjects learned at different rates from choice and no-choice outcomes, as the most parsimonious description of decision-making behaviour (Supplementary material). Learning rates for choice trials were larger than learning rates for no-choice trials for most (440 of 475, 93%) participants. These results indicate that whilst participants learned from all outcomes, they learned most from outcomes over which they had more control. However, there were no significant Spearman’s correlations between parameter estimates and mood scores (Supplementary material). We repeated this correlation analysis using a ‘default’ Bayesian hypothesis test and found strong evidence for the absence of a correlation between depressive symptom severity scores and each of the three parameters (1/30 < BF10 < 1/10) (Wetzels and Wagenmakers, 2012) (Supplementary material).

### General linear model voxel-based functional MRI analyses

As expected, across all 475 participants, significant reward activations were identified in areas including the ventral striatum (−12, 10, −14) (10, 8, −10), ventromedial prefrontal cortex (−4, 52, −10), orbitofrontal cortex (−24, 34, −20) (30, 34, −16) and mPFC (−10, 28, 0), as well as activations in the occipital lobe visual areas (10, −86, −6) (−10, −88, −8). As hypothesized, significant negative correlations between reward activation magnitude and QIDS mood symptom scores were found in areas including the striatum (8, 10, 16) (−10, 8, 22) (−16, 12, 4), and also in the insula (−34, 18, −12) (30, 16, −16) and dorsomedial prefrontal cortex (−4, 30, 50). Additionally, a positive correlation with QIDS scores was found in the occipital lobe (10, −86, 20) (−8, −90, 10).

A conjunction analysis of correlations with group-level activations and deactivations was done revealing that higher depressive symptoms were associated with decreased activation in the ventral striatum, and increased deactivation in caudate/dorsal striatum, anterior insula and dorsomedial PFC. As expected, results of RPE signal encoding correlations were similar (Supplementary material) because of a correlation between the RPE signal and simple binary reward outcome signals. Notably though, higher depressive symptoms were associated with decreased RPE signal encoding (only) in two additional areas: midbrain/ventral tegmental area (−2, −16, −16) and mPFC (−2, 34, 14). These results are shown in Fig. 2.

Across participants, reward activations were significantly larger during choice compared to no-choice conditions in regions including mPFC (0, 52, 16), insula (36, 18, −12) (−28, 18, −14) and amygdala (−22, −6, −14) (22, −4, −12) (Fig. 3). Higher depressive symptoms were also associated with decreased choice versus no-choice response difference in regions including the precuneus (0, −46, 36) and increased response difference in regions including left insula (−36, 16, 0) and subgenual anterior cingulate cortex (4, 28, 0). Additional results are presented in the Supplementary material.

### Dynamic causal modelling of event-related effective connectivity

The mean of the explained variance across the 165 participants with sufficiently strong functional MRI signals was 21.90%. There were no statistically significant differences in mean QIDS scores between the 165 participants with explained variance <10% (mean QIDS = 4.6) and excluded (mean QIDS = 4.4) participants (Welch’s *t*-test: *P *=* *0.618). Additional analyses showed that alternative variance thresholds led to similar DCM results (Supplementary material).

**Figure 4:**
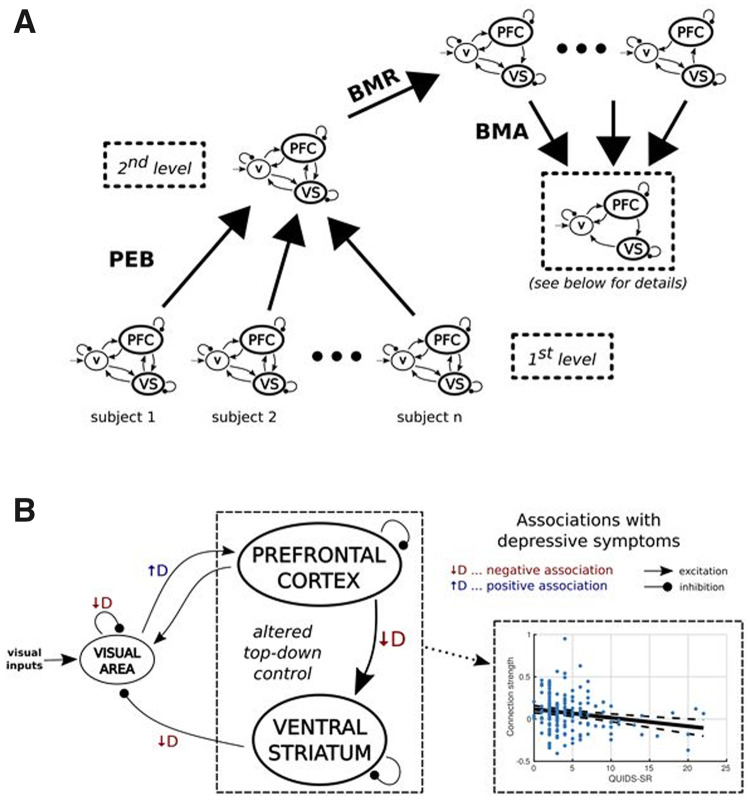
**Effective connectivity analyses**. (**A**) Individual DCMs were taken to the second level where Bayesian model reduction (BMR) was performed to ‘prune’ connections. Bayesian model averaging (BMA) was then used to average the DCMs, weighted by their probabilities. (**B**) Top-down control of the prefrontal cortex over the ventral striatum (VS) was decreased with increasing depression symptom severity.

At the group level and consistent with known anatomy, there was insufficient evidence for effective connectivity from the visual area to ventral striatum and from ventral striatum to mPFC, but all other connections had a high probability. Details about group-commonalities are presented in the Supplementary material; here we focus on associations with QIDS mood symptoms. Of particular interest was the directed influence (connectivity) from the mPFC to the ventral striatum. This was found to be negatively correlated with mood symptom scores (Fig. 4B); higher depression scores were related to a decreased top-down mPFC to ventral striatum influence. Notably, we did not find this association with anxiety scores (Supplementary material). The analyses also revealed complicated indirect interactions between the visual cortex and both cortical and subcortical regions. Specifically, higher depressive symptoms were negatively associated with the connection from the accumbens and positively associated with the connection to the mPFC.

Additional control analyses were carried out to verify that our results did not depend on the exact specifications of the model, covariates and variance threshold criteria. The negative association between depressive symptom severity and directed influence from the mPFC to ventral striatum was found for each analysis strategy (Supplementary material).

We investigated the association of connection strengths with individual symptoms assessed with QIDS (rather than the sum-of-scores) and also performed this analysis after excluding current and past MDE participants. This exploratory analysis revealed a more specific negative association between symptoms of ‘concentration or decision making difficulties’ and the top-down connection from mPFC to ventral striatum and this result was replicated after a stepwise exclusion of current MDE and past MDE participants (Supplementary material).

### Bootstrap split-sample replication of mPFC to ventral striatum effective connectivity correlation

**Figure 5:**
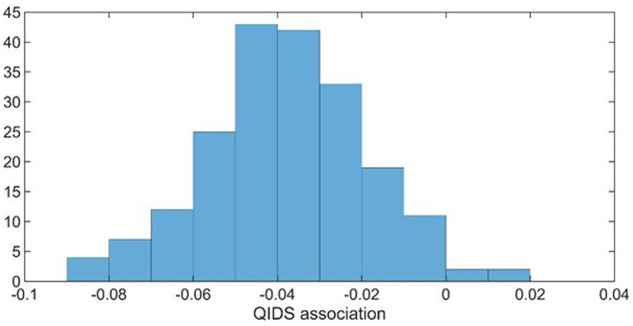
**Association between QIDS and mPFC to ventral striatum effective connectivity.** Histogram after 100 random splits in the total data.

The dataset was randomly split into two halves repeatedly and the second level PEB model (without Bayesian model reduction) estimated for each half. Figure 5 shows the histogram of results for 100 splits (200 second level models) of the association between QIDS and mPFC to ventral striatum effective connectivity. The association was negative in 98% of cases, showing the QIDS blunted effective connectivity result can be replicated on a split-sample basis.

## Discussion

Data from a large community-based study was used to test hypotheses that mood symptoms were associated with blunted reward signal. Increased depressive symptoms were indeed found to be negatively correlated with reward-linked signals in the striatum, consistent with many independent studies (Forbes *et al.*, 2006; Steele *et al.*, 2007; Kumar *et al.*, 2008, 2018; Pizzagalli *et al.*, 2009; Eshel and Roiser, 2010; Gradin *et al.*, 2011; Zhang *et al.*, 2013; Pizzagalli, 2014; Johnston *et al.*, 2015; Rothkirch *et al.*, 2017; Keren *et al.*, 2018) and large community-based studies that used reward anticipation during a monetary incentive delay task, which did not include a decision-making component (Stringaris *et al.*, 2015; Pornpattananangkul *et al.*, 2019). We also tested whether individuals learned differently from outcomes depending on whether they had control over decisions. Subjects did learn differently from outcomes depending on whether they had control over their decisions that lead to the outcomes; however, we did not find a clear influence of depressive symptoms. Matched behaviour but differences in brain function might indicate a compensatory mechanism. Future studies should consider the possibility that depression might also alter the interaction between cortico-limbic connectivity and task-related events such as choice versus no-choice reward outcomes.

Previous independent clinical studies have reported RPE abnormalities in MDD (Kumar *et al.*, 2008, 2018; Gradin *et al.*, 2011; Dombrovski *et al.*, 2013). Here we found decreased RPE signal encoding in many of the same striatal areas as decreased reward responses. It is difficult to disentangle RPE encoding from a binary reward signal (Chowdhury *et al.*, 2013) in our task as the signals are correlated, which is common. However, we also found the ventral tegmental area was associated with an RPE (but not binary) signal, which was negatively correlated with mood score, consistent with an independent study on treatment-resistant depression (Gradin *et al.*, 2011). The ventral tegmental area is strongly implicated in the brain’s reward system and RPE signals (Schultz *et al.*, 1997) and is a source of dopaminergic projections to the ventral striatum and frontal cortex (Alexander and Crutcher, 1990; Alexander *et al.*, 1990). Reduced reward-linked effective connectivity from the ventral tegmental area to striatum has been reported (Kumar *et al.*, 2018).

There is substantial evidence for resting state connectivity abnormalities in MDD (Kaiser *et al.*, 2015, 2016), indicating that this illness is not only associated with abnormalities in isolated brain regions, but also interactions between these brain regions. Recent reviews and meta-analyses point towards widespread network dysfunction in MDD but much of the work has focused on undirected functional connectivity or connectivity measured during the resting state. Notably, a recent functional connectivity study using resting state functional MRI reported decreased cingulo-striatal connectivity in children related to anhedonia (Pornpattananangkul *et al.*, 2019).

Here we identified significant directed mPFC to striatal reward-linked effective connectivity. This projection has been reported to consist of excitatory glutamatergic neurons from studies on animals (Alexander and Crutcher, 1990; Alexander *et al.*, 1990). A glutamatergic hypothesis of depression has been proposed (Sanacora *et al.*, 2012) and reduced glutamatergic projections from the prefrontal cortex to the striatum have been argued to underlie motivational disorders such as addiction (Kalivas, 2009) and depression (Russo and Nestler, 2013). Indeed, many prominent theories of MDD propose a role for abnormal cortical-limbic connectivity such as Beck’s cognitive model (Disner *et al.*, 2011), Mayberg’s cortical-limbic dysregulation model (Mayberg, 1997), Pizzagalli’s stress interaction model (Pizzagalli, 2014) and Roiser’s neuropsychological model (Roiser *et al.*, 2012). Different abnormalities have been reported for the medial prefrontal cortical region. Mayberg’s deep brain stimulation was applied to the subgenual cingulate Brodmann area 25, which they found to be overactive in depression using long timescale PET imaging (Mayberg *et al.*, 2005), while reward-related activity in the medial prefrontal region used in our connectivity model has been reported to be decreased using short timescale event-related functional MRI (Johnston *et al.*, 2015).

We also found evidence for changes in effective connectivity between the visual processing area and both striatum and mPFC. Alterations in these indirect connections are more difficult to interpret and we did not have strong *a priori* hypotheses about them. It is notable that the connection from the striatum to the visual region may occur via the amygdala (Dolan, 2002; Amaral *et al.*, 2003), a region strongly implicated in depression, so it is possible that the back projection from the amygdala to early visual areas is affected by depression.

Decreased accumbens activity may be due to decreased cortical to striatal connectivity, consistent with prominent theories of depression. Most (95%) accumbens neurons are GABAergic medium spiny neurons, so a decrease in blood oxygen level-dependent (BOLD) activity during functional MRI may reflect a change in the inhibitory output from the accumbens, with opposite effects for D1-type direct pathway versus D2-type indirect pathway neurons (Russo and Nestler, 2013). Reward-gain tasks are controlled by activation of the D1-type direct pathway and punishment-avoidance tasks by inactivation of the D2-type indirect pathway (Nakanishi *et al.*, 2014). Here the task was reward-gain similar to our previous independent clinical case-control studies (Steele *et al.*, 2007; Gradin *et al.*, 2011; Johnston *et al.*, 2015) although we have also reported punishment-avoidance accumbens abnormalities in treatment-resistant MDD (Johnston *et al.*, 2015). Striatal BOLD activation in the present study may therefore predominately have reflected activation of D1-type direct pathway medium spiny neurons, and blunting of this signal with depressive symptoms impairment of the direct pathway.

The strengths of this work are between-study replication of blunted reward-linked striatal signals in a large community-based sample and the novel finding of blunted mPFC to striatal event-related connectivity, which was replicated on a split-sample within-study basis. There are, however, some limitations as potential avenues for future work. During both computational and imaging analyses a common model was assumed for all participants although in principle, models could differ between subjects (Stephan *et al.*, 2009). DCM is a region of interest approach and we chose our regions to test for hypothesized differences between activated regions. Future studies should consider tasks that activate additional regions, such as the amygdala and hippocampus (Mayberg, 1997; Drevets *et al.*, 2008; Roiser *et al.*, 2012; Pizzagalli, 2014; Johnston *et al.*, 2015; Schmaal *et al.*, 2015) and explore trans-diagnostic (Cuthbert and Insel, 2013) constructs such as anhedonia. We did not have a hypothesis about abnormal connectivity in one hemisphere compared to another. To maximize the number of included subjects we focused on the hemisphere that had the strongest signals across subjects. Exploration of possible covariates was done to determine whether our conclusions about a significant negative association could be confounded by such effects, not because we had specific hypotheses about these. Our conclusions were unchanged (Supplementary material). To make an unbiased estimate of cortico-limbic connectivity it was necessary to include subjects who had sufficient signals to allow valid estimation, despite depression being associated with reward-signal blunting. When this was done a significant negative association with depression severity was found, which was not dependent on the precise criteria used for selecting data. Including all subjects, even those with the weakest signals, resulted in connectivity estimates being dominated by noise, although a non-significant negative trend remained (Supplementary Fig. 8). Importantly, none of the analyses suggested significantly increased cortico-limbic connectivity (Supplementary material). We did not have a specific hypothesis about which sub-symptom of depression would be associated with altered cortical-to-subcortical connectivity and note that our finding of an association with concentration or decision-making difficulties will need to be independently replicated.

In conclusion, using an RDoC positive valence system approach with a large community-based sample, we found evidence that depressive symptom severity was related to blunting of reward-linked striatal activity, consistent with a series of case-control studies on MDD. Decreased striatal activity may be due to decreased cortical-to-striatal event-related effective connectivity consistent with prominent theories of depression, and here have resulted in decreased direct pathway basal ganglia output. Further study of basal ganglia pathophysiology is required to better understand these abnormalities and develop new treatments.

## Supplementary Material

awaa106_Supplementary_Data

## Abbreviations

DCM =: dynamic causal modelling
MDD =: major depressive disorder
mPFC =: medial prefrontal cortex
QIDS =: Quick Inventory of Depressive Symptomatology
RPE =: reward prediction error
