# Supplementary material for: Blunted medial prefrontal cortico-limbic reward-related effective connectivity and depression
Source: Brain. 2020 May 8;143(6):1946–56. doi: 10.1093/brain/awaa106 (PMC7296844; doi:10.1093/brain/awaa106)
Supplement: awaa106_Supplementary_Data [file awaa106_supplementary_data.zip › awaa106-suppl_data/brain-2019-02224-File007.pdf]

Supplementary Material

Blunted Medial Prefrontal Cortico-Limbic Reward-Related Effective Connectivity and Depression

Samuel Rupprechter[1], Liana Romaniuk[2], Peggy Series[1], Yoriko Hirose[2], Emma Hawkins[2], Anca-Larisa Sandu[3], Gordon D. Waiter[3], Christopher J. McNeil[3], Xueyi Shen[2], Mathew A. Harris[2], Archie Campbell[4], David Porteous[4], Jennifer A. Macfarlane[5], Stephen M. Lawrie[2], Alison D. Murray,[3] Mauricio R. Delgado[6], Andrew M. McIntosh[2], Heather C. Whalley [2]*, J. Douglas Steele[5]*

[1]School of Informatics, University of Edinburgh, UK; [2]Division of Psychiatry, University of Edinburgh; [3]Biomedical Imaging Centre, University of Aberdeen; [4]Centre for Genomic and Experimental Medicine, University of Edinburgh, [5]Division of Imaging Science and Technology, Medical School, University of Dundee, [6]Department of Psychology, Rutgers University. *joint last authors

Participant Details

Table 1 (in the main text) and Table S23 contain details about participants' demographics and clinical information. Diagnostic screening showed that our sample included 20 participants satisfying criteria for MDD, 110 participants matching remitted MDD, and 345 never-depressed subjects. Dynamic Causal Modelling (DCM) of event-related connectivity was done with data from an initial 301 participants (after excluding participants with insufficient signal in our regions of interest, 19 MDE remained, who were further filtered to include subjects for whom the explained variance of the full DCM for each subject exceeded variable minimum thresholds (see DCM section below). This is summarised in Figure S11.

QIDS scores covered a wide range (0 to 23), although the distribution was skewed (Figure S1). Additional non-parametric Spearman's rank correlation tests were used to test for the relationship between QIDS and reward signals to minimize the risk of outliers affecting our mass-univariate GLM results (see below). Figure S2 shows a histogram of the number of missed trials.

Figure S1. Histogram of participants' QIDS scores.

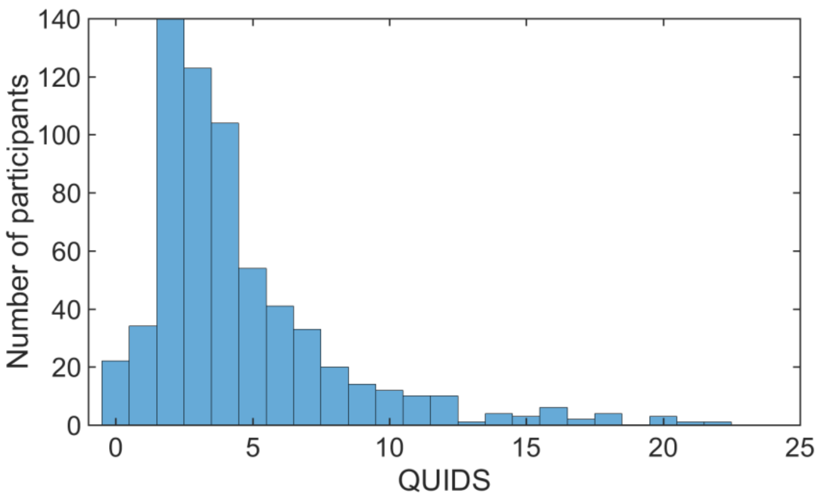

Figure S2. Histogram of number of missed trials.

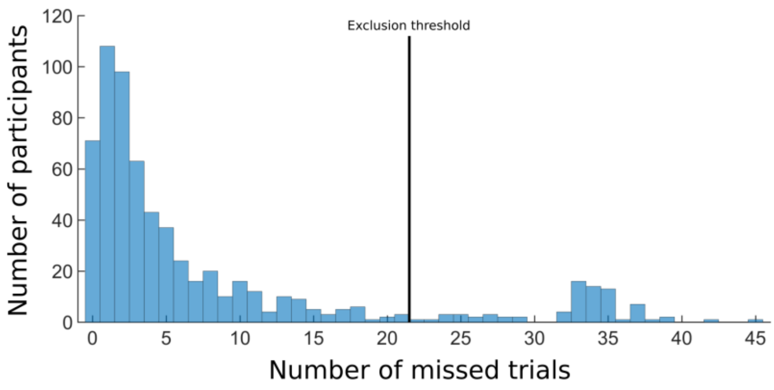

Participants with more than 21 missed trials were excluded (77 participants).

Computational modelling

Table S1. Model specification.

Name	Description	Value update: $V^{(t+1)}$	Parameters
M1	only learn from choice condition	$V^{(t)} + \varepsilon_c \times \left(r^{(t)} - V^{(t)}\right)$	ε_c, β
M2	learn equally from choice/no-choice conditions	$V^{(t)} + \varepsilon \times \left(r^{(t)} - V^{(t)}\right)$	ε, β
M3	learn differently from choice/no-choice conditions	$V^{(t)} + \varepsilon_D^{(t)} \times \left(r^{(t)} - V^{(t)}\right)$	$\varepsilon_c, \varepsilon_n, \beta$
M4	experience reward differently during choice/no-choice conditions	$V^{(t)} + \varepsilon \times \left(\rho_D^{(t)} \times r^{(t)} - V^{(t)}\right)$	$\varepsilon, \rho_c, \rho_n$
M5	learn and experience reward differently during choice/no-choice conditions	$V^{(t)} + \varepsilon_D^{(t)} \times \left(\rho_D^{(t)} \times r^{(t)} - V^{(t)}\right)$	$\varepsilon_c, \varepsilon_n, \rho_c, \rho_n$

The third column shows how internal values are updated after observing an outcome r in trial t. Choices were modelled probabilistically by passing the value difference to a logistic sigmoid function: p(choose V_1*) = 1 / (1 + e^(-β (*$V_1 - V_2$*))). ε is the learning rate with c and n being indicators for separate choice/no-choice learning rates; β is the inverse temperature parameter; ρ is the reward sensitivity parameter with c and n being indicators for separate choice/no-choice parameters.*

Model-fitting and model comparison

Parameter estimation (for each model) followed a hierarchical procedure. For each participant, we first estimated maximum likelihood (ML) estimates and then combined these into a group prior (normal distribution). The prior was then used to estimate maximum *a posteriori* (MAP) parameter values for each participant. These estimates were again combined into a single group prior and the procedure was iterated until convergence. We used the integrated Bayesian information criteria (iBIC) to perform model comparison. Sampling was used to estimate an integral over parameters, which was used to approximate the model evidence. iBIC scores were computed for each model and compared to choose the most parsimonious model. More details are available (Supplements of Huys et al. 2013 and Rupprechter et al. 2018).

Computational modelling results

Model comparison identified *Model 3* as the most parsimonious model (Figure S3) and subsequent analyses only focussed on this model. For each participant we calculated the asymptotic internal value estimations for the two stimuli as the average over the last 10 trials. The results are depicted in Figure S4, which shows that participants' value estimations are close to the actual probabilities of the two stimuli. The two estimated learning rate parameters of the winning model were highly correlated across participants (Pearson's r=0.883, $p<10^{-10}$) but parameter recovery simulations showed they could both be recovered. Spearman's correlations were calculated between QIDS scores and each the three model parameters. No correlation was significant (choice learning rate: Spearman's ρ=0.046, p=0.316; no-choice learning rate: Spearman's ρ=0.056, p=0.226; inverse temperature parameter: Spearman's ρ=0.076, p=0.098). We then performed a "default Bayesian hypothesis test" (Wetzels & Wagenmakers, 2012) which allowed us to quantify evidence *for* the null hypothesis of no correlation. This relies on estimated Pearson correlations which were all similar to the Spearman's correlations and non-significant (choice learning rate: r=0.032, p=0.487; no-choice learning rate: r=0.047, p=0.302; inverse temperature parameter: r=0.017, p=0.704). The estimate Bayes factors were: choice learning rate: BF10=0.047, no-choice learning rate: BF10=0.062, inverse temperature parameter: BF10=0.039. These values can be interpreted as "strong" evidence in favour of the null (Wetzels & Wagenmakers, 2012).

Whilst Huys et al. (2013) found meta-analytic evidence for an association of depressive symptom scores (specifically anhedonia) and a "reward sensitivity" model parameter, which is closely related to our "inverse temperature" model parameter, we did not find this effect. Differences between studies could account for this. It is important to note that we used an overall depressive symptoms score instead of a specific

anhedonia score. Additionally the signal detection task used by Huys et al. involves many more trials and the influence of reward sensitivity on behaviour might only become apparent after many trials.

F*igure S3. Model comparison results.*

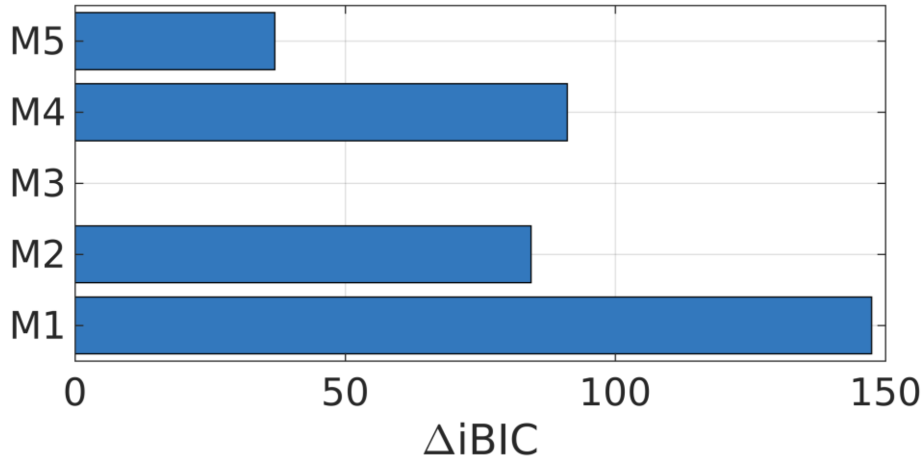

iBIC scores relative to the lowest (i.e. best) iBIC score are shown. M3 which includes different learning rates depending on choice or no-choice condition was the most parsimonious model. M1 assumed participants did not learn from no-choice trials. M2 assumed participants learned equally well from both conditions (same learning rate parameter). M4 assumed that it was reward responsiveness that was dependent on condition but not learning rate (one learning rate parameter, two reward sensitivity parameters). M5 assumed both learning rate and reward sensitivity were dependent on condition.

Figure S4. Asymptotic internal value estimations.

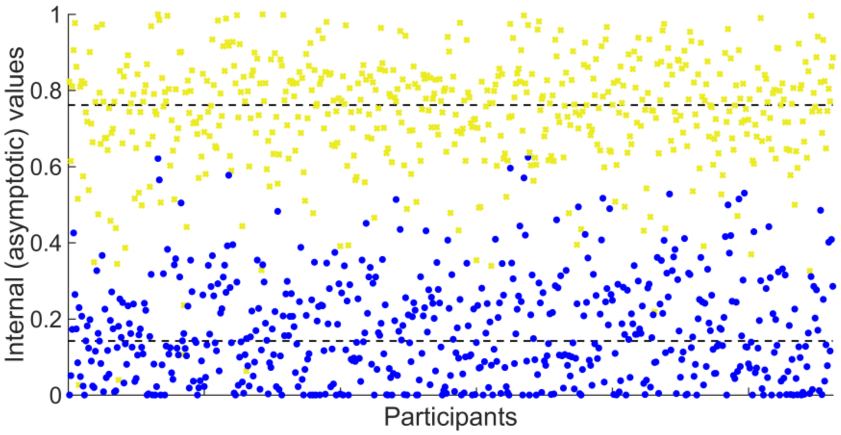

Yellow dots are asymptotic value estimations for the yellow stimulus (80% reward probability) and blue dots are asymptotic value estimations for the blue stimulus (20% reward probability). The black dotted lines show the average asymptotic value estimations across participants for the two stimuli, which are close to the true reward probabilities.

Neuroimaging analyses

Pre-processing details

SPM12 (version 7487) was used for analyses. Functional images were manually checked for artefacts before pre-processing. The first six blood oxygen level-dependent volumes were discarded as standard because of transient effects. Functional images were realigned to the first image using a rigid body spatial transformation (6 parameters) and unwarped. The estimated movement parameters were plotted and manually inspected for excessive motion. If such excessive motion was identified, the corresponding functional images were inspected and participants were excluded if there were noticeable excessive movement. Slice timing correction was performed with the middle slice as the reference slice. (We therefore did not use the additional slice timing correction model included as part of DCM.) The T1 weighted structural image was segmented using SPM12 tissue probability maps and the ICBM space template for European brains and functional images were co-registered to the bias corrected T1 image. The estimated deformation field was then used to spatially normalise the images and an 8 mm FWHM Gaussian kernel was used to smooth the normalised images. The registration was inspected manually using SPM's checkreg tool and participants were excluded if the registration quality was judged insufficient.

Freesurfer ROIs

Raw T1 images were segmented and parcellated using FreeSurfer version 5.3 (Dale et al., 1999; Fischl et al., 1999; Fischl et al., 2004) and the Desikan-Killany atlas (Desikan et al., 2006). FreeSurfer output was visually quality checked, major errors were excluded and minor errors were corrected manually. To create ROI masks, FreeSurfer parcellations were reoriented to the original image and converted back to NIfTI format. Using FSL 5.0 (Smith et al., 2004; Woolrich et al., 2009; Jenkinson et al., 2012), these images were thresholded to isolate each ROI and then binarised to create a mask. ROIs were normalised in the same way as functional images using the estimated deformation field.

Multiple comparisons correction

To correct for multiple comparisons we used Monte Carlo simulations (Slotnick & Schacter, 2004) to establish a cluster extent threshold. This relies on the fact that the larger a cluster the less likely it is that each individual voxel in the cluster shows spurious activity and survives an individual voxel threshold. The script we used can be downloaded from the author's website (https://www2.bc.edu/sd-slotnick/scripts.htm) who also defended this method in subsequent publications (Slotnick, 2017). The parameters we used are as follows: x_matrix=64; y_matrix=64; slices=32; dim_xy=3.4; dim_z=4.5; mask_name='none'; mask_bytes=0; mask_plot=0; FWHM=8; dim_resampled=2; iterations=5000; p_corrected=0.001; p_voxel=0.05. Note that in Tables S2-S9 we usually list a small number of local maxima at least 8mm apart but we focus on interpreting clusters of activity rather than individual voxels.

Signal dropout

For group level analyses, SPM only includes voxels which are included in the mask of every individual. Signal dropout of voxels within a single participant therefore excludes those voxels from further analyses for all participants, which can become a problem when a large number of participants are involved. For first level analyses the masking threshold was therefore lowered to 0.4 to increase the included area. An explicit mask was used to constrain analysis to voxels within the brain (SPM's intracranial volume mask; mask_ICV.nii). Nevertheless, a few areas were excluded due to signal dropout (Figure S5), including parts of the right VS and a region in the PFC including subgenual ACC and OFC.

Figure S5. Group-level signal dropout.

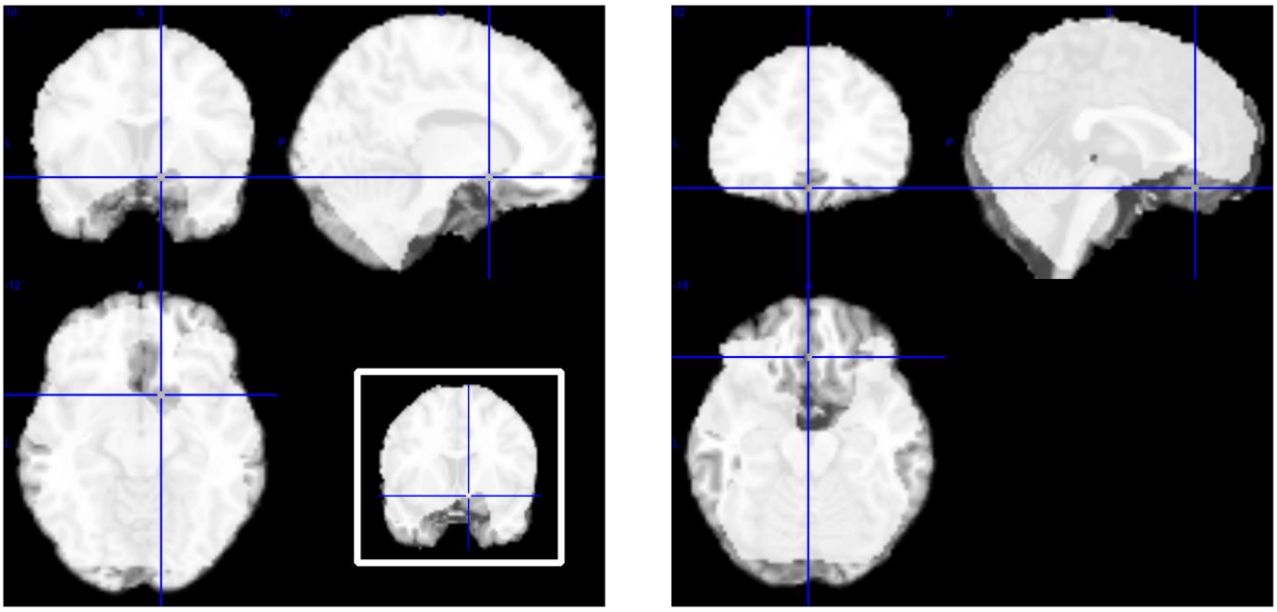

The second level mask (white) is shown as overlay on a Colin brain. **Left**: Dropout in the right VS (12,10,-12); the inset of the bottom right shows a small part of the right VS which is included in the mask (10,8,-10). **Right**: Dropout in the subgenual ACC and OFC (0,32,-18).

fMRI results tables

Table S2. Reward activation across participants.

Reward > No-reward (p<0.05 FWE correction)					
k	T	x	y	z	
19270	40.05	10	-86	-6	Occipital lobe
	38.91	-20	-80	-14	
	38.43	-10	-88	-8	
710	15.38	-52	0	48	Premotor cortex L
	6.77	-40	-2	62	
426	12.12	-16	44	50	dPFC
	5.89	-32	34	48	
	5.47	-22	22	64	
1311	10.97	-58	-8	-12	Temporal gyrus L
	9.66	-58	-30	0	
	7.97	-50	-40	2	
3036	10.61	-12	10	-14	Ventral striatum, vmPFC
	9.95	-4	54	-10	
	9.87	-6	66	16	
237	9.18	-22	-30	-2	Hippocampus L
117	7.91	54	38	6	PFC
	6.76	52	42	-2	
	5.80	56	34	16	
48	7.80	22	42	50	PFC
	6.23	14	50	46	
177	7.62	-26	-56	52	Parietal lobe L
97	7.04	24	-56	52	Parietal lobe R
73	6.56	24	-28	-2	Hippocampus R
122	5.91	-28	-8	2	Putamen L
82	5.68	-28	-8	-22	Hippocampus L
79	5.47	62	0	-14	Temporal lobe R
	5.33	58	-6	-18	
3	5.32	46	50	2	PFC
3	4.97	16	38	58	PFC
19	4.96	-22	-42	-48	Cerebellum
3	4.93	6	58	38	
2	4.86	-4	54	42	
3	4.75	20	16	22	
3	4.75	28	48	36	
4	4.72	62	18	26	
1	4.66	58	8	-18	
1	4.63	32	36	48	
1	4.58	-24	14	-24	
1	4.57	-26	18	-24	

Table S3. Reward deactivation across participants.

No-reward > Reward (p<0.05 FWE correction)					
k	T	x	y	z	
4938	13.22	42	-72	42	Angular gyrus R
	13.00	58	-56	34	
	6.82	64	-52	-8	
6654	12.54	0	22	50	SMA, dPFC / MCC
	11.99	12	16	62	
	10.40	42	14	46	
1156	11.39	46	16	0	Insula R
	9.65	34	22	0	
2925	10.17	-58	-62	28	Angular gyrus L
	9.59	-58	-50	44	
	9.56	-62	-46	38	
787	9.92	-40	18	2	Insula L
	9.28	-30	22	-8	
1347	8.62	4	-62	48	Precuneus
	7.72	8	-66	64	
192	7.36	-12	6	6	Caudate L
333	7.23	22	54	18	PFC
	6.29	32	52	8	
153	6.73	-32	-58	-32	Cerebellum
	5.75	-24	-72	-32	
	4.85	-14	-78	-30	
87	6.28	14	6	6	Caudate R
159	6.18	-30	52	16	PFC
16	5.41	-44	-60	-46	cerebellum
15	5.15	48	-4	-36	Temporal lobe R

Table S4. Effects of choice reward outcomes.

Reward (choice) > Reward (no-choice) (p<0.05 FWE correction)					
k	T	x	y	z	
1290	8.76	36	18	-12	Insula R
	7.43	46	26	-2	
	5.41	34	20	-28	
2394	8.50	0	52	16	mPFC / rostral ACC
	6.96	4	42	26	
	6.46	18	58	28	
259	6.83	-28	18	-14	Insula L
165	6.20	-32	-92	6	Occipital lobe
65	5.33	-22	-6	-14	Amygdala L
142	5.32	-34	-82	-10	Occipital lobe
	4.97	-30	-74	-14	
74	5.26	22	-4	-12	Amygdala R
	5.13	22	6	-8	
5	4.75	-12	4	-10	VS L

Table S5. Negative association between depressive severity and reward outcome signals in areas of reward activation across participants.

Conjunction analysis (each p<0.001 whole brain cluster corrected): (1) negative association of reward outcome signal with QIDS (2) *activation* across participants during reward outcome					
k	T	x	y	z	
3580	3.50	26	4	0	Putamen R + L, OFC
	3.20	32	-2	6	
	2.99	-26	18	-20	
168	2.51	-6	-60	-32	Cerebellum
	2.37	-6	-52	-34	
	2.13	-16	-44	-40	

Table S6. Negative association between depressive severity and reward outcome signals in areas of reward deactivation across participants.

Conjunction analysis (each p<0.001 whole brain cluster corrected): (1) negative association of reward outcome signal with QIDS (2) *deactivation* across participants during reward outcome					
k	T	x	y	z	
1629	4.01	-16	10	6	Caudate L, insula L
	4.00	-34	18	-10	
	3.65	-14	8	16	
478	3.84	18	8	6	Caudate R
1727	3.38	-4	30	50	dmPFC, ACC
	3.23	10	32	48	
	2.57	24	56	20	
162	3.22	-36	22	46	PFC L
	2.00	-34	16	36	
260	3.17	-42	-62	-48	Cerebellum
	2.79	-38	-62	-36	
860	2.74	-58	-46	34	Angular gyrus L
	2.40	-60	-58	18	
	2.28	-52	-56	30	
459	2.47	28	24	-6	Insula R
	2.30	28	20	10	
	2.27	54	6	14	
198	2.38	42	24	44	PFC R
	2.12	36	20	40	
	2.05	32	14	30	

Table S7. Positive association between depressive severity and reward outcome signals in areas of reward activation across participants.

Conjunction analysis (each p<0.001 whole brain cluster corrected): (1) positive association of reward outcome signal with QIDS (2) *activation* across participants during reward outcome					
k	T	x	y	z	
1403	3.13	10	-86	20	Occipital lobe
	2.92	4	-82	2	
	2.84	-8	-90	10	

Table S8. Negative association between depressive severity and reward prediction error signals in areas of positive RPE signals across participants.

Conjunction analysis (each p<0.001 whole brain cluster corrected): (1) negative association of RPE signal with QIDS (2) *positive RPE signal encoding* across participants					
k	T	x	y	z	
3304	3.98	32	-2	6	Putamen R + L, pallidum R + L, OFC R,
	3.87	22	-2	2	midbrain / VTA
	3.30	32	-14	4	
	2.26	8	-16	-10	
327	3.10	-42	12	-30	Temporal lobe L
	2.40	-34	4	-30	
	2.23	-36	6	-22	
198	2.89	-28	-62	-46	Cerebellum L
	2.16	-38	-64	-46	
	1.71	-38	-62	-38	
872	2.58	16	36	10	ACC
	2.55	-10	38	22	
	2.40	14	42	24	
161	2.53	-34	22	-16	OFC L
	2.47	-26	20	-20	
	1.88	-50	28	-16	

Table S9. Negative association between depressive severity and reward prediction error signals in areas of negative RPE signals across participants.

Conjunction analysis (each p<0.001 whole brain cluster corrected): (1) negative association of RPE signal with QIDS (2) *negative RPE signal encoding* across participants					
k	T	x	y	z	
632	3.14	46	14	-2	Insula R
	2.94	46	12	6	
	2.83	56	6	6	
384	2.86	-34	12	12	Insula L
	2.69	-42	10	2	
	2.39	-38	16	-4	
160	2.72	-12	10	2	Caudate L
	2.02	-16	20	2	
143	2.58	-40	-62	14	
324	2.54	-4	28	52	dmPFC
	2.39	10	32	48	
	2.28	-4	38	48	
165	2.28	-52	-56	32	Parietal lobe
	2.10	-54	-58	42	

Control analyses

We repeated our correlation analyses using a GLM with additional covariates “site”, “sex”, and “age” (mean-centred). We also performed additional control analysis excluding 103 participants who were related to another participant (372 remaining). The results of these analyses were very similar to the results in Tables S2-S9 and here we only show that the negative association between depressive severity and reward signals in the striatum (Figure S6).

Figure S6. Correlations with depressive symptom scores.

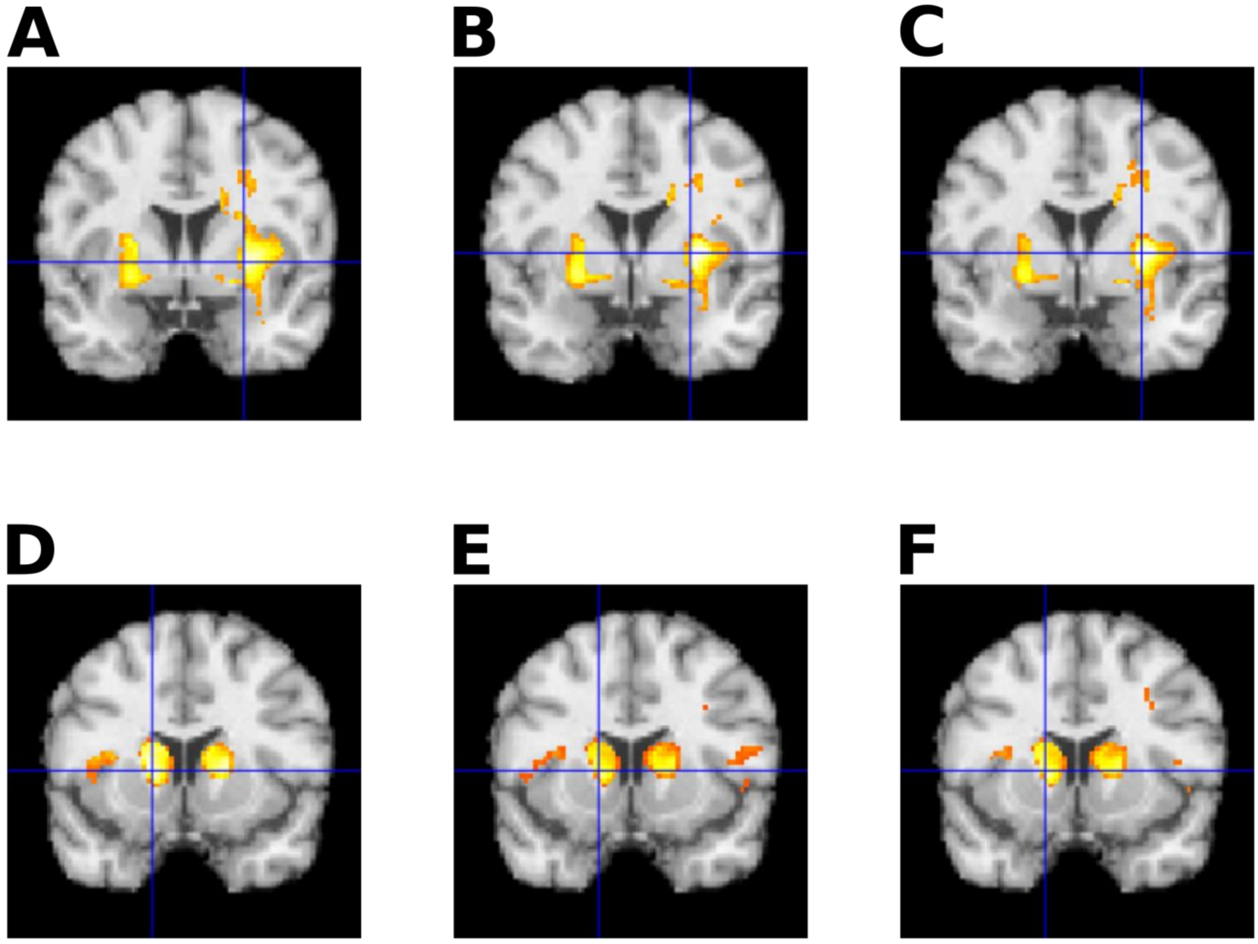

This shows the results of conjunction analyses of activation or deactivation across participants and negative association with depressive symptoms (see also Figure 2 in the main text). (A and D) Results following the inclusion of additional covariates site, sex and age. (B and E) Results following the exclusion of related participants. (C and F) Results following exclusion of related participants and inclusion of additional covariates.

Non-parametric correlations

Additional non-parametric Spearman correlation tests were performed between QIDS scores and median contrast (*reward > baseline*) beta values extracted of 4mm spheres centred around local maxima of the GLM analyses (Table S10). The results agree with the previous analysis, with the exception that the positive association between depressive symptoms scores and reward signal encoding in the occipital lobe was not significant.

Table S10. Spearman rank correlation analysis between reward signals and QIDS scores.

x	y	z	Spearman's ρ	p	
18	8	6	-0.150	0.001	basal ganglia
26	4	0	-0.159	0.0005	putamen
32	-2	6	-0.127	0.005	putamen
-24	6	6	-0.152	0.0009	putamen
20	6	-6	-0.127	0.006	ventral striatum
-12	10	2	-0.121	0.008	ventral striatum
-16	10	6	-0.198	1.34e-05	caudate
-4	30	50	-0.156	0.0006	medial PFC
16	36	10	-0.127	0.006	rostral ACC
-34	18	-10	-0.183	6.15e-05	insula
4	-82	2	0.089	0.054	occipital lobe
10	-86	20	0.071	0.124	occipital lobe
-8	-90	10	0.044	0.337	occipital lobe

The median estimated beta values from individuals' contrast files were extracted from voxels within 4mm spheres centred around coordinates of significant reward signals. Correlations were calculated between these beta values and depressive severity scores and results are presented here.

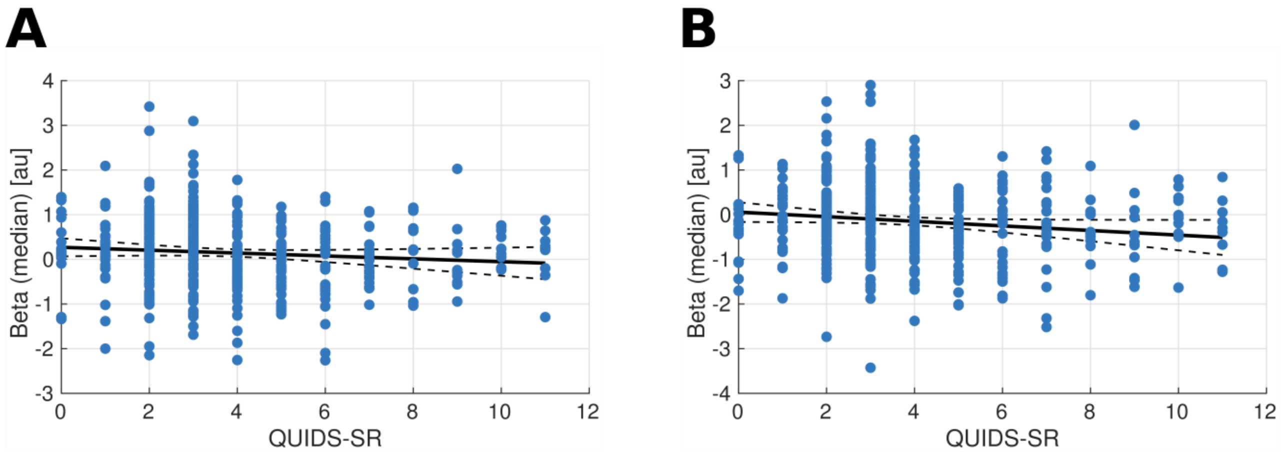

Figure S7. Scatter plot of QIDS scores and median reward-contrast beta estimates.
Participants with QIDS scores larger than 11 were excluded and correlations remained significant after these exclusions. (A) 4mm spheres centred at (26,4,0); Spearman's $\rho=-0.140$, $p=.003$. (B) 4mm sphere centred at (-16,10,6); Spearman's $\rho=-0.180$, $p<.001$.

DCM Analysis

Dynamic causal modelling (DCM12.5; Friston et al. 2003) was used to test for effective (directed) interactions between brain regions. DCM treats the brain as a deterministic non-linear dynamic system which receives inputs and then produces outputs. The unknown (arbitrary) function describing the evolution of the neuronal states is parameterised using a bilinear approximation, which reduces the parameters to three important sets: (a) the interaction between neuronal systems in the absence of input ("A" matrix), (b) the change of this interaction induced by (experimentally-controlled) inputs ("B" matrix), (c) extrinsic experimentally-controlled inputs ("C" matrix). A haemodynamic model (containing additional parameters which are usually not of interest) is used to map the underlying neuronal state changes to the haemodynamic response (i.e. the observed BOLD signal).

Time-series extraction

For the time-series extraction a GLM was used containing columns for outcomes, reward outcomes, choice outcomes, choice cues, no-choice cues, choice responses, no-choice responses and nuisance regressors, and an "effects of interest" F contrast was computed (including all effects except nuisance regressors). ROIs were defined as a combination (logical AND) of the following ROIs: (a) 12mm spheres centred around coordinates of (group-level) local maxima (see main-text), (b) subject-specific whole brain mask (*mask.nii* from first level analysis), (c) thresholded SPM mask using a liberal threshold (p<0.15; note that this was used to exclude noisy voxels and was not used to infer statistical significance) for the *reward* (visual area and VS) or choice (mPC) contrast, (d) anatomical FreeSurfer masks for the visual area (pericalcarine) and VS (striatum). Time-series were extracted from each of these (combined) ROIs, adjusted for the effects of interest. We were not able to extract time-series from all three regions for 174 participants (because in at least one region there were no voxels in the combined mask).

Variance thresholds

We used a script included with SPM (spm_dcm_fmri_check.m) to calculate the proportion of explained signal by the model. As suggested in the function's documentation and the SPM mailing-list (https://www.jiscmail.ac.uk/cgi-bin/webadmin?A0=SPM), we set an *a priori* threshold at 10% and excluded participants below this threshold. Examples of models with low variance explained were inspected manually and many of these models seemed to have "flat-lined", meaning estimates did not converge from their prior mean.

Control analyses

We performed several control analyses to verify that our results did not depend on the exact specification of the model. First, we increased the threshold of the minimum explained variance to 15% and repeated the PEB analysis with the remaining 99 participants (mean variance explained 28.31%). Similarly, we reduced the threshold to 7.5% and repeated the analysis with 208 participants (mean variance explained 19.20%) and also computed the top-down connection strength as a function of the threshold (see *Variance threshold* section below). Second, we repeated the analysis without including covariates and with including only anxiety (HADS-A) as covariate. Third, we estimated a single PEB model for both "A" and "B" DCM matrices (54 parameters; only using QIDS and site as covariates to limit the dilution of evidence). Last, we re-defined the experimentally-controlled inputs as outcome(choice, reward), outcome(choice, no reward), outcome(no-choice, reward), outcome(no-choice, no-reward) in addition to the (unchanged) inputs cue(choice), cue(no choice), response(choice) and response(no choice). It was again assumed that each of the four outcome conditions could modulate each of the endogenous connections.

Results of control analyses are displayed in Tables S12-S17. Although the exact values of estimated parameters varied and automatic pruning did not always identify the exact same connections as important,

there was generally a large overlap between the individual results. Importantly, the negative association between QIDS depressive severity and intrinsic connectivity strength from mPC to VS remained for *every* single analysis strategy. Note that any parameter not pruned during the automatic search is important and useful in that it contributes to the free energy, even if the posterior probability is less than 95%.

PEB-BMA results: intrinsic connections (A matrix)

Tables S11-S17 list posterior probabilities and effect sizes of intrinsic connections related to commonalities and differences across participants. Results for different variance thresholds are shown and with various covariates included. The connection from mPC to VS is highlighted in all tables. It is the only connection that has non-zero probability of there being an effect of QIDS for every single analysis strategy.

Table S11. BMA results of intrinsic connections.

	P(common)	P(QIDS)	P(HADS-A)	P(site)	P(age)	P(sex)	P(MDD)
A(1,1)	1	1	0	1	0.72	0	0
A(2,1)	1	1	0	0.62	0	0	0
A(3,1)	0	0	0	0.51	0	0	1
A(1,2)	1	0	0	0.56	0	0	0
A(2,2)	1	0	1	0	1	0	0
A(3,2)	1	1	0	1	0	1	0
A(1,3)	1	1	0.73	0.8	0.63	0	0
A(2,3)	0	0	0	1	0	1	0
A(3,3)	1	0	1	0	0	0.66	0

	E(common)	E(QIDS)	E(HADS-A)	E(site)	E(age)	E(sex)	E(MDD)
A(1,1)	0.480	-0.019	0.000	0.152	-0.006	0.000	0.000
A(2,1)	0.121	0.013	0.000	0.021	0.000	0.000	0.000
A(3,1)	0.000	0.000	0.000	0.013	0.000	0.000	0.145
A(1,2)	0.450	0.000	0.000	-0.032	0.000	0.000	0.000
A(2,2)	-0.369	0.000	0.031	0.000	0.006	0.000	0.000
A(3,2)	0.065	-0.011	0.000	-0.048	0.000	0.086	0.000
A(1,3)	-0.232	-0.030	-0.017	0.060	0.004	0.000	0.000
A(2,3)	0.000	0.000	0.000	-0.095	0.000	0.135	0.000
A(3,3)	-0.245	0.000	-0.013	0.000	0.000	-0.054	0.000

165 participants; 10% variance threshold; P(x) shows the posterior probability of the estimates (E(x)) being non-zero. The first column identifies the connection as A(TO, FROM), where 1=visual area, 2=mPC, 3=VS; e.g. A(1,2) is the connection from mPC to visual area

Table S12. BMA results of intrinsic connections using an increased quality threshold.

	P(common)	P(QIDS)	P(HADS-A)	P(site)	P(age)	P(sex)	P(MDD)
A(1,1)	1	1	0	1	0.54	0	1
A(2,1)	1	1	0	0.71	0	0	0
A(3,1)	0.73	0.71	1	1	0	1	0.7
A(1,2)	1	0	0	1	0.53	0	0
A(2,2)	1	1	0	0	0	0	0
A(3,2)	1	1	0	1	0	1	0
A(1,3)	1	0.77	1	1	0	0	0
A(2,3)	1	0	0	0.77	0	1	0
A(3,3)	1	0	1	0	1	1	0

	E(common)	E(QIDS)	E(HADS-A)	E(site)	E(age)	E(sex)	E(MDD)
A(1,1)	0.424	-0.032	0.000	0.122	-0.003	0.000	0.596
A(2,1)	0.135	0.017	0.000	0.028	0.000	0.000	0.000
A(3,1)	-0.024	0.009	-0.016	0.059	0.000	-0.077	0.107
A(1,2)	0.429	0.000	0.000	-0.085	0.003	0.000	0.000
A(2,2)	-0.316	0.022	0.000	0.000	0.000	0.000	0.000
A(3,2)	0.068	-0.010	0.000	-0.082	0.000	0.109	0.000
A(1,3)	-0.179	-0.018	-0.033	0.088	0.000	0.000	0.000
A(2,3)	0.114	0.000	0.000	-0.052	0.000	0.178	0.000
A(3,3)	-0.311	0.000	-0.020	0.000	0.006	-0.169	0.000

99 participants; 15% variance threshold; P(x) shows the posterior probability of the estimates (E(x)) being non-zero. The first column identifies the connection as A(TO, FROM), where 1=visual area, 2=mPC, 3=VS; e.g. A(1,2) is the connection from mPC to visual area.

Table S13. BMA results of intrinsic connections using a decreased quality threshold.

	P(common)	P(QIDS)	P(HADS-A)	P(site)	P(age)	P(sex)	P(MDD)
A(1,1)	1	1	0	1	0	0	0
A(2,1)	1	1	0	0	0	0	0
A(3,1)	0.73	0	0	0.51	0	0	1
A(1,2)	1	0.63	0	0.56	0	0	0
A(2,2)	1	0	1	0	1	0	0
A(3,2)	1	1	0	1	0	0	0
A(1,3)	1	1	0.76	0.59	0.51	0	0
A(2,3)	0	0	0	1	0	0.65	0
A(3,3)	1	0	1	0	0	1	0

	E(common)	E(QIDS)	E(HADS-A)	E(site)	E(age)	E(sex)	E(MDD)
A(1,1)	0.539	-0.021	0.000	0.157	0.000	0.000	0.000
A(2,1)	0.104	0.010	0.000	0.000	0.000	0.000	0.000
A(3,1)	-0.021	0.000	0.000	0.012	0.000	0.000	0.153
A(1,2)	0.436	-0.009	0.000	-0.031	0.000	0.000	0.000
A(2,2)	-0.361	0.000	0.033	0.000	0.005	0.000	0.000
A(3,2)	0.058	-0.010	0.000	-0.039	0.000	0.000	0.000
A(1,3)	-0.247	-0.027	-0.018	0.034	0.003	0.000	0.000
A(2,3)	0.000	0.000	0.000	-0.082	0.000	0.066	0.000
A(3,3)	-0.243	0.000	-0.012	0.000	0.000	-0.110	0.000

208 participants; 7.5% variance threshold; P(x) shows the posterior probability of the estimates (E(x)) being non-zero. The first column identifies the connection as A(TO, FROM), where 1=visual area, 2=mPC, 3=VS; e.g. A(1,2) is the connection from mPC to visual area.

Table S14. BMA results of intrinsic connections using a PEB matrix without covariates.

	P(common)	P(QIDS)	E(common)	E(QIDS)
A(1,1)	1	0.96	0.471	-0.023
A(2,1)	1	0.98	0.121	0.012
A(3,1)	0	0	0.000	0.000
A(1,2)	1	0	0.447	0.000
A(2,2)	1	1	-0.366	0.023
A(3,2)	1	0.76	0.069	-0.007
A(1,3)	1	1	-0.237	-0.042
A(2,3)	0	0	0.000	0.000
A(3,3)	1	0	-0.244	0.000

165 participants; 10% variance threshold; P(common) and P(QIDS) show the posterior probability of the estimates (E) being non-zero. The first column identifies the connection as A(TO, FROM), where 1=visual area, 2=mPC, 3=VS; e.g. A(1,2) is the connection from mPC to visual area.

Table S15. BMA results of intrinsic connections using a PEB matrix with a single covariate (anxiety).

	P(common)	P(QIDS)	P(HADS-A)	E(common)	E(QIDS)	E(HADS-A)
A(1,1)	1	0.95	0	0.475	-0.023	0.000
A(2,1)	1	0.97	0	0.120	0.012	0.000
A(3,1)	0	0	0	0.000	0.000	0.000
A(1,2)	1	0	0	0.449	0.000	0.000
A(2,2)	1	0	1	-0.366	0.000	0.031
A(3,2)	1	0.78	0	0.069	-0.008	0.000
A(1,3)	1	0.92	0.82	-0.236	-0.027	-0.022
A(2,3)	0	0	0	0.000	0.000	0.000
A(3,3)	1	0	0.7	-0.245	0.000	-0.008

165 participants; 10% variance threshold; P(common), P(QIDS), and P(HADS-A) show the posterior probability of the estimates (E) being non-zero. The first column identifies the connection as A(TO, FROM), where 1=visual area, 2=mPC, 3=VS; e.g. A(1,2) is the connection from mPC to visual area.

Table S16. BMA results of intrinsic connections using an alternative input specification.

	P(common)	P(QIDS)	E(common)	E(QIDS)
A(1,1)	1	0	0.674	0.000
A(2,1)	0.98	0	0.050	0.000
A(3,1)	0.62	0.93	-0.015	0.010
A(1,2)	1	0	0.453	0.000
A(2,2)	1	0	-0.402	0.000
A(3,2)	1	0.98	0.071	-0.014
A(1,3)	0	0	0.000	0.000
A(2,3)	0.7	0	0.042	0.000
A(3,3)	1	0	-0.396	0.000

141 participants; 10% variance threshold; common variance explained = 19.89%; P(common) and P(QIDS) show the posterior probability of the estimates (E) being non-zero. The first column identifies the connection as A(TO, FROM), where 1=visual area, 2=mPC, 3=VS; e.g. A(1,2) is the connection from mPC to visual area. See Control analyses section for input specification.

Table S17. BMA results of intrinsic connections using an alternative input specification and anxiety as covariate.

	P(common)	P(QIDS)	P(HADS-A)	E(common)	E(QIDS)	E(HADS-A)
A(1,1)	1	0	0.62	0.677	0.000	0.012
A(2,1)	0.98	0	0	0.051	0.000	0.000
A(3,1)	0.64	1	0.46	-0.016	0.012	-0.004
A(1,2)	1	0	0.93	0.457	0.000	0.023
A(2,2)	1	0.66	1	-0.401	-0.013	0.024
A(3,2)	1	1	0	0.072	-0.015	0.000
A(1,3)	0	0	0	0.000	0.000	0.000
A(2,3)	0.71	0	0	0.046	0.000	0.000
A(3,3)	1	0	0.57	-0.397	0.000	-0.008

141 participants; 10% variance threshold; mean variance explained = 19.89%; P(common), P(QIDS) and P(HADS-A) show the posterior probability of the estimates (E) being non-zero. The first column identifies the connection as A(TO, FROM), where 1=visual area, 2=mPC, 3=VS; e.g. A(1,2) is the connection from mPC to visual area.

PEB results: modulations (B matrix)

Tables S18-S21 list posterior probabilities and effect sizes of modulations of connections related to commonalities and differences (QIDS) across participants. Table S21 lists posterior probabilities and effect sizes of a control analysis for which a single PEB model was defined for both intrinsic connectivity and modulations.

Table S18. BMA results of modulations by reward outcome conditions.

	P(common)	P(QIDS)	E(common)	E(QIDS)
B(1,1)	1	0	-1.346	0.000
B(2,1)	0	0	0.000	0.000
B(3,1)	0	0	0.000	0.000
B(1,2)	0	0.82	0.000	-0.069
B(2,2)	0.98	0.88	-0.579	-0.068
B(3,2)	0	0	0.000	0.000
B(1,3)	0	0	0.000	0.000
B(2,3)	0.62	0	0.185	0.000
B(3,3)	1	0	-0.775	0.000

P(common) and P(QIDS) show the posterior probability of the estimates (E) being non-zero. The first column identifies the modulation of connection as B(TO, FROM), where 1=visual area, 2=mPC, 3=VS; e.g. B(1,2) is the modulation of the connection from mPC to visual area.

Table S19. BMA results of modulations by reward omission conditions.

	P(common)	P(QIDS)	E(common)	E(QIDS)
B(1,1)	0	0	0.000	0.000
B(2,1)	0	0	0.000	0.000
B(3,1)	0	0	0.000	0.000
B(1,2)	0	0	0.000	0.000
B(2,2)	1	0	-0.957	0.000
B(3,2)	0	0	0.000	0.000
B(1,3)	0.88	0.99	0.492	0.187
B(2,3)	0	0	0.000	0.000
B(3,3)	1	0	-1.223	0.000

P(common) and P(QIDS) show the posterior probability of the estimates (E) being non-zero. The first column identifies the modulation of connection as B(TO, FROM), where 1=visual area, 2=mPC, 3=VS; e.g. B(1,2) is the modulation of the connection from mPC to visual area.

Table S20. BMA results of modulations by choice outcome conditions.

	P(common)	P(QIDS)	E(common)	E(QIDS)
B(1,1)	1	0	-0.626	0.000
B(2,1)	1	0	-0.238	0.000
B(3,1)	1	0	0.251	0.000
B(1,2)	0.99	0	-0.519	0.000
B(2,2)	0.98	0	-0.615	0.000
B(3,2)	0	0	0.000	0.000
B(1,3)	0	0	0.000	0.000
B(2,3)	0	0	0.000	0.000
B(3,3)	1	1	-0.959	0.114

Bayesian model average results. P(common) and P(QIDS) show the posterior probability of the estimates (E) being non-zero. The first column identifies the modulation of connection as B(TO, FROM), where 1=visual area, 2=mPC, 3=VS; e.g. B(1,2) is the modulation of the connection from mPC to visual area.

Table S21. BMA results of modulations by no-choice outcome conditions.

	P(common)	P(QIDS)	E(common)	E(QIDS)
B(1,1)	1	0	-0.527	0.000
B(2,1)	1	0	-0.180	0.000
B(3,1)	1	0	0.147	0.000
B(1,2)	1	0	-0.660	0.000
B(2,2)	1	0	-0.916	0.000
B(3,2)	0	0	0.000	0.000
B(1,3)	0	0	0.000	0.000
B(2,3)	0	0	0.000	0.000
B(3,3)	1	0	-1.050	0.000

P(common) and P(QIDS) show the posterior probability of the estimates (E) being non-zero. The first column identifies the modulation of connection as B(TO, FROM), where 1=visual area, 2=mPC, 3=VS; e.g. B(1,2) is the modulation of the connection from mPC to visual area.

Table S22. Control analysis BMA results of intrinsic connections and modulations

		P(mean)	P(QIDS)	P(site)	E(mean)	E(QIDS)	E(site)
	A(1,1)	1	0.71	1	0.441	-0.011	0.147
	A(2,1)	1	1	0	0.123	0.012	0.000
	A(3,1)	1	0	0.58	-0.033	0.000	0.014
	A(1,2)	1	0	0.55	0.443	0.000	-0.030
	A(2,2)	1	1	0	-0.457	0.027	0.000
	A(3,2)	1	0.72	1	0.069	-0.006	-0.045
	A(1,3)	1	1	1	-0.230	-0.039	0.092
	A(2,3)	0	0	1	0.000	0.000	-0.091
	A(3,3)	1	0	0	-0.376	0.000	0.000
reward	B(1,1)	1	0	1	-1.115	0.000	-0.361
	B(2,1)	0	0	0	0.000	0.000	0.000
	B(3,1)	0	0	0	0.000	0.000	0.000
	B(1,2)	0	1	1	0.000	-0.098	-0.381
	B(2,2)	1	0	1	-1.166	0.000	0.417
	B(3,2)	0	0	1	0.000	0.000	0.177
	B(1,3)	0	0	0	0.000	0.000	0.000
	B(2,3)	0	0	0	0.000	0.000	0.000
	B(3,3)	1	0	1	-1.179	0.000	0.383
reward omission	B(1,1)	0.52	1	0	-0.169	0.074	0.000
	B(2,1)	0	0	0	0.000	0.000	0.000
	B(3,1)	0	0	0	0.000	0.000	0.000
	B(1,2)	0	0	0	0.000	0.000	0.000
	B(2,2)	1	0	1	-1.155	0.000	0.463
	B(3,2)	0	0	0	0.000	0.000	0.000
	B(1,3)	0	1	1	0.000	0.216	0.487
	B(2,3)	0	0	0	0.000	0.000	0.000
	B(3,3)	1	0	0	-1.340	0.000	0.000
choice	B(1,1)	1	0	0	-0.906	0.000	0.000
	B(2,1)	1	0	0	-0.122	0.000	0.000
	B(3,1)	1	0	0	0.106	0.000	0.000
	B(1,2)	0	0	0	0.000	0.000	0.000
	B(2,2)	1	0	0	-1.175	0.000	0.000
	B(3,2)	0	0	0	0.000	0.000	0.000
	B(1,3)	0	0	0	0.000	0.000	0.000
	B(2,3)	0	0	0	0.000	0.000	0.000
	B(3,3)	1	1	0	-1.210	0.087	0.000
no-choice	B(1,1)	1	0	0.62	-0.845	0.000	-0.122
	B(2,1)	1	0	0	-0.105	0.000	0.000
	B(3,1)	0.52	0	0	0.038	0.000	0.000
	B(1,2)	1	0	0	-0.309	0.000	0.000
	B(2,2)	1	0	0	-1.174	0.000	0.000
	B(3,2)	0	0	0	0.000	0.000	0.000
	B(1,3)	0	0	0	0.000	0.000	0.000
	B(2,3)	0	0	0	0.000	0.000	0.000
	B(3,3)	1	0.59	0	-1.297	-0.038	0.000

(See Tables S12 and S21 for legends.)

Summary of main DCM results

Modulation of connectivity

On average across subjects, reward outcomes modulated visual area, mPC and VS self-connectivity. Self-inhibition was decreased during reward outcomes leading to increased overall activation in these regions (as observed during model-free GLM analysis). No difference in modulation was associated with QIDS scores. Similarly, no-reward outcomes modulated self-connectivity of mPC and VS. Self-inhibition was decreased leading to overall increased activation. Both mPC and VS showed stronger negative modulation during no-reward than during reward outcomes (leading to decreased inhibition and therefore stronger activation during no-choice outcomes). In addition, differences in the no-reward modulation of the VS to the visual area (inhibitory) connection were positively associated with QIDS scores. This means higher depressive symptoms were associated with increased excitation / decreased inhibition from VS to the visual area.

Outcomes during both choice and no-choice conditions modulated the following connections: visual area→visual area, visual area→mPC, visual area→VS, mPC→visual area, mPC→mPC, VS→VS. Self-inhibition was decreased for all regions, leading to increased activation. Self-inhibition of the visual area was stronger and self-inhibition of VS and mPC was weaker during choice outcomes compared to no-choice outcomes. Visual area to VS connectivity was increased during both choice and no-choice outcomes, and higher during choice compared to no-choice outcomes. Choice and no-choice outcome conditions decreased the connectivity from the visual area and mPC and from mPC to the visual area. Compared to choice outcomes, no-choice outcomes had a weaker negative modulation effect on the visual area→mPC connection, but a stronger negative modulation effect on the mPC→visual area connection. Higher depressive symptom scores were associated with increased modulation of VS self-inhibition during choice outcomes only. This means that while outcomes from participants' own choice overall led to an increase in VS activity (through decreased inhibition), in participants with higher depressive symptoms this increase was reduced.

Summary of DCM results organised by region

Visual area: There was evidence for effective (excitatory) endogenous connectivity to mPC, which was increased with higher depressive symptoms. The region's self-inhibition was weaker in participants with higher depressive symptoms. The medial PFC region had an excitatory influence on the visual area, while VS had an inhibitory influence and the connection strength was negatively associated with depressive symptoms. Choice and no-choice outcomes were associated with increased influence on VS. Self-inhibition was weaker (resulting in increased activity) during all outcomes except no-reward outcomes. Both choice and no-choice conditions decreased the connectivity to and from mPC. Higher depressive symptoms were associated with decreased inhibition from VS during no-reward outcomes.

mPC: There was evidence for excitatory endogenous connections to and from the visual area and to VS. The excitation from the visual area and the mPC self-inhibition increased with higher depressive symptoms. The influence of mPC on VS decreased with higher depressive symptoms. Self-inhibition was weaker during all outcomes compared to baseline. Both choice and no-choice outcomes decreased the connectivity to and from the visual area.

VS: Received excitatory input from mPC and its activity exerted inhibitory influence on the visual area. The influence from mPC was decreased with higher depressive symptoms. The connection strength to the visual area was negatively associated with depressive symptoms. Self-inhibition was weaker during all outcomes compared to baseline. Choice and no-choice outcomes were associated with increased influence of the visual area, with stronger modulation during choice than no-choice outcomes. Higher depressive symptoms were associated with increased influence on the visual area during no-reward outcomes and stronger self-inhibition during choice outcomes.

Variance threshold

In Figure S8 we plot the association of QIDS with VS to VS connectivity as a function of the variance threshold. It shows the expected values (c.f. E(QIDS) of A(3,2) in previous tables) of the second level PEB model (without BMR). Covariates included (zero-mean centred) QIDS, HADS anxiety, site age and sex. As expected, it roughly follows an inverted U shape. At low thresholds noise suppresses the association and at high thresholds too few participants remain.

Figure S8. Association of top-down connection strength with QIDS as a function of variance threshold.

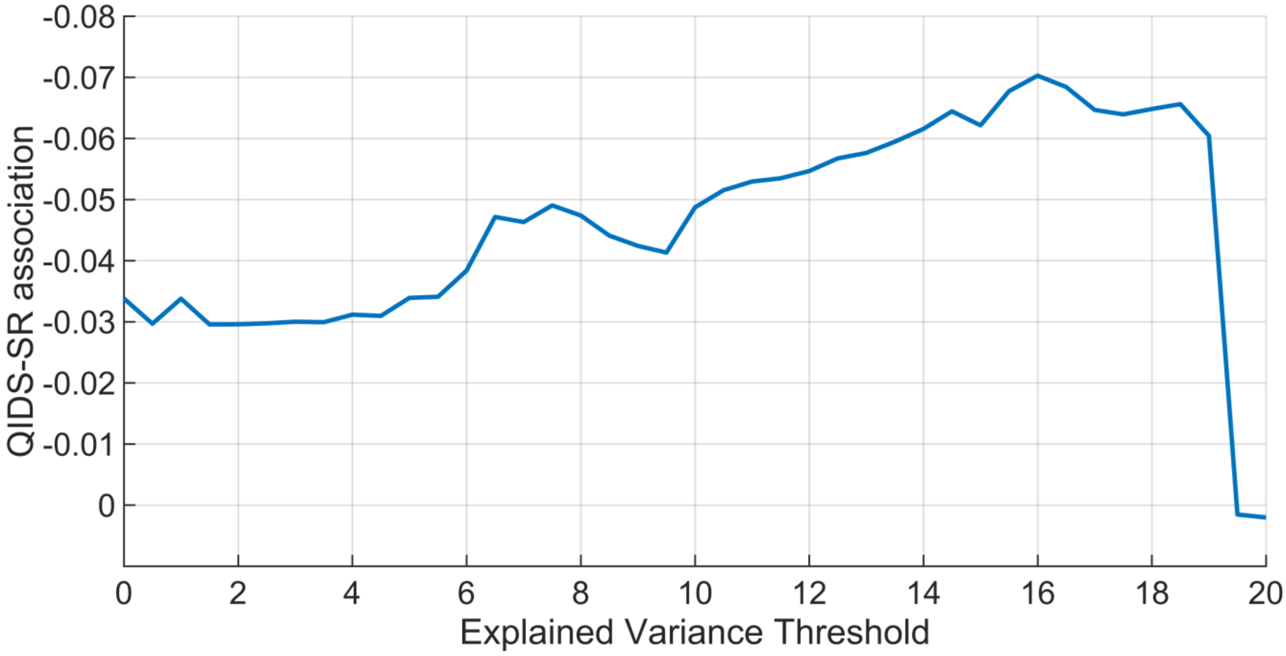

The association of the covariate of interest (QIDS) with the connection from VS to accumbens as a function of the variance threshold in the estimated PEB model. As described earlier, our a priori chosen threshold was 10%.

For the variance threshold we followed DCM recommendations. To make an unbiased estimate of connectivity it was necessary to include data from subjects who had sufficient signals. To convince ourselves of the validity of our findings we performed a large number of control analyses. Importantly, there was no significant difference in depression symptom severity between included and excluded participants, making it extremely unlikely that exclusions biased our main results.

We varied the DCM threshold criterion, including up to 301 participants (see Table S23), and observed exactly what we predicted (Figure S8): at very low thresholds the reward activation signals are so low that noise dominates which suppresses the negative association, although a non-significant negative trend remains when all subjects were included. (At very high thresholds so few subjects were included that the statistical power was affected and the association was not significant). In Tables S12-13 we show the results of another two full BMA analyses using higher and lower thresholds with very similar results to the main text analysis. Finally, we show that the negative association also holds within random splits of the data (Figure 5).

Consequently we believe it is extremely unlikely that the exact number of inclusions biased our main result, which is the association between depression symptom severity and effective cortico-limbic connectivity. Rather than lowering the variance threshold post-hoc, which would have allowed us to include more participants, we chose to be consistent with DCM recommendations on signal threshold to allow valid inferences, so used additional control analyses to provide an unbiased estimate of abnormal connectivity.

In summary, when we used data with sufficient signals to make valid inferences we found a significant negative association between cortico-limbic connectivity and depressive symptoms, when there was insufficiently strong signals we couldn’t draw conclusions, and none of the analyses suggested a significant positive relationship between cortico-limbic connectivity and depressive symptoms.

Analyses of individual depression symptoms

There are some potential issues of the correlation analysis we performed so far. QIDS scores are skewed and correlation results might be affected by the heteroscedasticity. Closely related to this, we performed the analysis across groups and since the MDD group naturally displayed higher depressive symptom scores, it is possible our results related more closely to a group effect rather than an effect of increasing symptom severity (although we did try to account for that by including an additional group-indicator covariate). Finally, the sum of the 16 individual symptom scores of our depression questionnaire might hide additional variation related to individual symptoms (Fried & Nesse, 2015).

To begin to address these potential concerns, we performed additional analyses for which we included each individual QIDS symptom (i.e. question) in the PEB design matrix. In the questionnaire each question was coded as a number from 0 to 3 with increasing severity. For the design matrix columns were transformed to code absence (score=0) or presence (score>0) of the symptom and not mean centered so that the mean column corresponded to a participant without any symptoms and each symptom column coded the additive effect of having the symptom. Other covariates (anxiety, age, sex, site) were again zero-mean centered. This means we essentially performed a list of ‘group comparisons’ of ‘participants reporting a specific symptom’ (e.g. concentration/decision-making difficulties) versus ‘participants who did not report this symptom’.

We also repeated this analysis after excluding current MDD participants and then again after excluding both current MDD and remitted MDD participants. Results are shown in the additional Supplement Tables document). The top-down connection from the prefrontal cortex to the accumbens was related to the presence of a number of symptoms, most notably “concentration or decision making difficulties” which was found in a variety of different analyses strategies including the analysis which did not include past or present MDD participants. This means participants reporting changes in their usual capacity to concentrate or make decisions had a decreased top-down connectivity. We also found evidence for decreased top-down control in participants displaying changes in their general interest, a substantial sub-part of “anhedonia”, but this was only true when all participants were included. Importantly however, only 4 never-depressed participants reported “general interest” symptoms, but 18 reported “concentration or decision making difficulties”. We note that these are exploratory analyses and we did not have strong hypotheses about which symptoms would be most associated with the connection from VS to VS.

As a final remark, it is worth mentioning that the symptom of decreased energy (one of the three core symptoms of a major depressive episode as defined in ICD-10 and also related to “motivation”) was associated with increased VS (self-)inhibition which would also lead to decreased activation as observed in many previous fMRI studies. In this study blunted reward response associated with *overall* depressive symptoms severity was mainly found in caudate and putamen. This might mean that blunting in different parts of the striatum could be related to (severity of) different depression symptoms.

Figure S11. Project overview and analysis workflow.

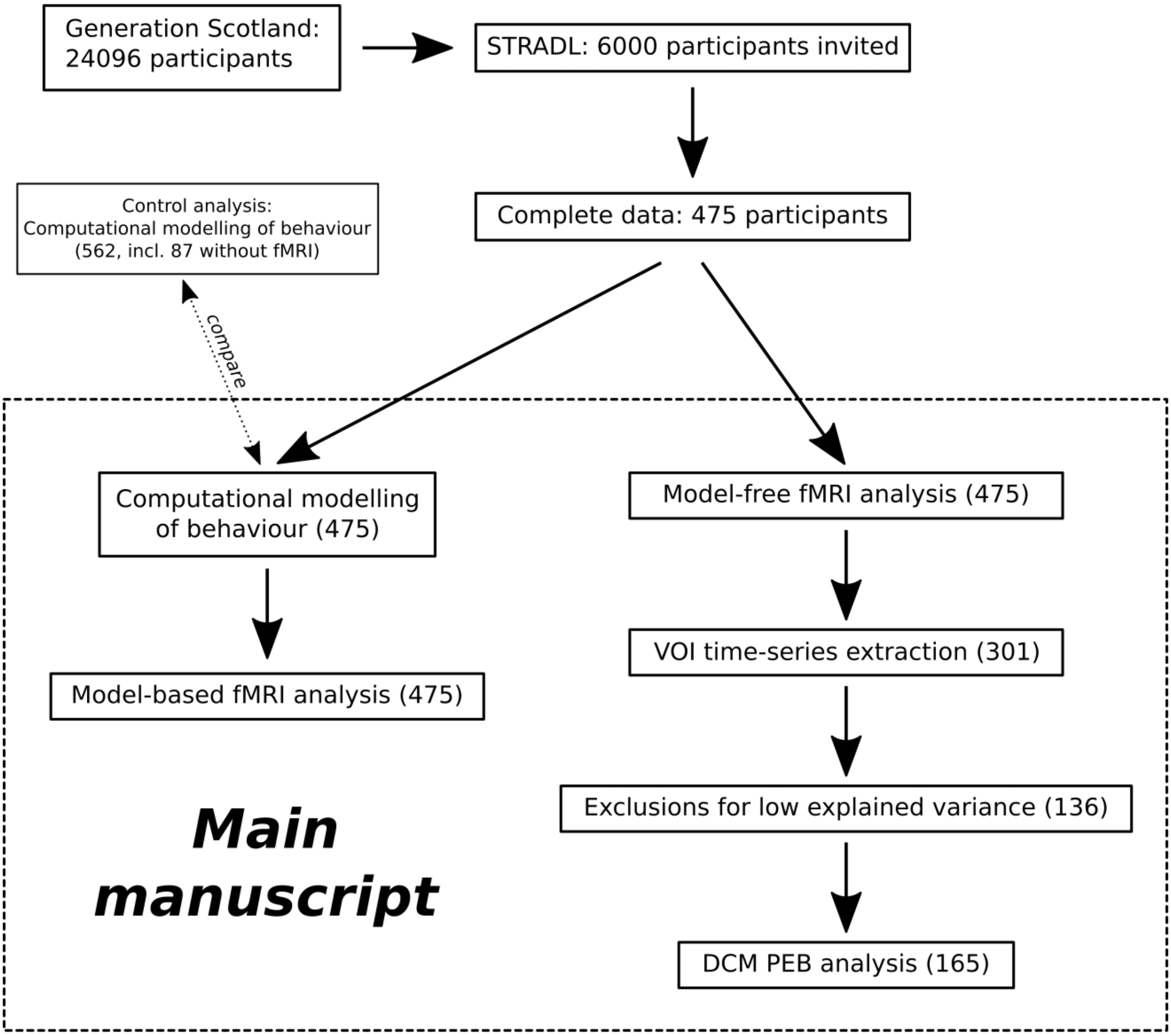

Table S23. Demographic and clinical details after exclusions (c.f. Figure S11)

		Controls	Past MDD	Current MDD
652 participants (excluding 3 of unknown group)	Number of subjects	463	156	30
	QIDS (range, mean ± std)	0 – 14,<br>3.59 ± 2.24	1 – 22,<br>5.42 ± 3.84	7 – 21,<br>13.9 ± 3.53
	HADS-A (range, mean ± std)	3.24 ± 2.53	5.17 ± 3.58	11.13 ± 3.83
475 participants	Number of subjects	407	129	26
	QIDS (range, mean ± std)	0 -14,<br>3.52 ± 2.23	1 – 22,<br>5.33 ± 3.77	7 – 21,<br>14.12 ± 3.78
	HADS-A (range, mean ± std)	0 – 15,<br>3.20 ± 2.56	0 – 17,<br>5.15 ± 3.55	6 – 20,<br>11.15 ± 3.82
475 participants (c.f. Table 1)	Number of subjects	345	110	20
	QIDS (range, mean ± std)	0 – 12,<br>3.39 ± 2.08	1 – 22,<br>5.41 ± 3.84	9 – 21,<br>14.55 ± 3.79
	HADS-A (range, mean ± std)	0 – 12,<br>3.13 ± 2.44	0 – 17,<br>5.04 ± 3.35	6 – 20,<br>10.65 ± 3.62
301 participants	Number of subjects	214	68	19
	QIDS (range, mean ± std)	0 – 12,<br>3.36 ± 2.07	1 – 22,<br>5.43 ± 3.85	9 – 21,<br>14.47 ± 3.88
	HADS-A (range, mean ± std)	0 – 12,<br>3.09 ± 2.51	0 – 17,<br>5.37 ± 3.46	6 – 20,<br>10.42 ± 3.56
165 participants	Number of subjects	112	41	12
	QIDS (range, mean ± std)	0 – 10,<br>3.21 ± 1.86	1 – 22,<br>5.73 ± 4.35	10 – 21,<br>14.00 ± 3.95
	HADS-A (range, mean ± std)	0 – 12,<br>3.08 ± 2.55	0 – 17,<br>5.32 ± 3.73	6 – 20,<br>11.50 ± 3.90

QIDS = Quick Inventory of Depressive Symptomatology (Self Report;) HADS = Hospital Anxiety and Depression Scale

Additional computational modelling

It was possible to fit our computational models to the behaviour of 562 participants as some participants were excluded for fMRI pre-processing reasons (see Figure S11). In our hierarchical fitting approach every included participant can potentially influence every other participant (by changing the empirical group prior) and model comparison might also be affected. We therefore repeated our model fitting and model comparison including all 562 participants. The winning model remained the same. For each approach we extracted the estimated parameters from the winning model for the included participants. All three parameters were nearly identical for all participants (Pearson's r>.99 for each parameter). We note that for model-based fMRI we used the estimated parameters (and simulated hidden variables) from the model which was fitted to all participants.

Model recovery simulations

To assess the strength of our model comparison results, we performed model recovery simulations by simulating data from one of the models and then fitting each model to the simulated data. Model comparison was then used to see if it correctly identified the model which simulated the data as "best-fitting" model. Each model was simulated 20 times using 562 participants and 66 trials to match our experimental data. Table S24 shows the results of these model recovery simulations. It can be seen that while simple models (including our winning model) were recovered well, there was too little data (per individual) to reliably support the recovery of the more complicated models against their simpler versions. Given the similarity of models 3 (our winning model) and 5 (which also includes separate reward sensitivity parameters), we ran additional analysis to compare these two models and see if our result of increased learning with increased control was also reproducible using a more complicated version of our winning model. There was a large significant correlation between the instrumental learning rates (Spearman's $\rho=0.553$, $p<10^{-10}$) and between the Pavlovian learning rates (Spearman's $\rho=0.601$, $p<10^{-10}$) of the two models. As in our winning model, analysis of the alternative model 5 showed that the large majority of participants had a higher learning rate for choice trials than for no-choice trials (553 of 562, 98%; model 3: 499 of 562 or 89%). We repeated these model recovery simulations with only the included 475 participants which again gave us very similar results (Table S25) and we also repeated the correlation analysis between QIDS and model parameters which led us to the same conclusions.

Table S24. Model recovery (475 participants)

		Best-fitting model				
**Simulating model**		**M1**	**M2**	**M3**	**M4**	**M5**
	**M1**	20	0	0	0	0
	**M2**	0	20	0	0	0
	**M3**	0	2	18	0	0
	**M4**	0	7	1	8	4
	**M5**	0	0	9	7	4

Table S25. Model recovery (475 participants)

		Best-fitting model				
**Simulating model**		**M1**	**M2**	**M3**	**M4**	**M5**
	**M1**	20	0	0	0	0
	**M2**	0	20	0	0	0
	**M3**	0	0	20	0	0
	**M4**	0	13	0	7	0
	**M5**	0	0	16	1	3

Supplementary References

Dale, A. M., Fischl, B., & Sereno, M. I. (1999). Cortical surface-based analysis: I. Segmentation and surface reconstruction. *Neuroimage*, 9(2), 179-194.

Desikan, R. S., Ségonne, F., Fischl, B., Quinn, B. T., Dickerson, B. C., Blacker, D., ... & Albert, M. S. (2006). An automated labeling system for subdividing the human cerebral cortex on MRI scans into gyral based regions of interest. *Neuroimage*, 31(3), 968-980.

Fischl, B., Sereno, M. I., & Dale, A. M. (1999). Cortical surface-based analysis: II: inflation, flattening, and a surface-based coordinate system. *Neuroimage*, 9(2), 195-207.

Fischl, B., Van Der Kouwe, A., Destrieux, C., Halgren, E., Ségonne, F., Salat, D. H., ... & Caviness, V. (2004). Automatically parcellating the human cerebral cortex. *Cerebral cortex*, 14(1), 11-22.

Fried, E. I., & Nesse, R. M. (2015). Depression sum-scores don't add up: why analyzing specific depression symptoms is essential. *BMC medicine*, 13(1), 72.

Friston, K. J., Harrison, L., & Penny, W. (2003). Dynamic causal modelling. *Neuroimage*, *19*(4), 1273-1302.

Huys, Q. J., Pizzagalli, D. A., Bogdan, R., & Dayan, P. (2013). Mapping anhedonia onto reinforcement learning: a behavioural meta-analysis. *Biology of mood & anxiety disorders*, *3*(1), 12.

Jenkinson, M., Beckmann, C. F., Behrens, T. E., Woolrich, M. W., & Smith, S. M. (2012). Fsl. *Neuroimage*, 62(2), 782-790.

Rupprechter, S., Stankevicius, A., Huys, Q. J., Steele, J. D., & Seriès, P. (2018). Major Depression Impairs the Use of Reward Values for Decision-Making. *Scientific reports*, *8*(1), 13798.

Slotnick, S. D., & Schacter, D. L. (2004). A sensory signature that distinguishes true from false memories. *Nature neuroscience*, 7(6), 664.

Slotnick, S. D. (2017). Cluster success: fMRI inferences for spatial extent have acceptable false-positive rates. *Cognitive neuroscience*, 8(3), 150-155.

Smith, S. M., Jenkinson, M., Woolrich, M. W., Beckmann, C. F., Behrens, T. E., Johansen-Berg, H., ... & Niazy, R. K. (2004). Advances in functional and structural MR image analysis and implementation as FSL. *Neuroimage*, 23, S208-S219.

Wetzels, R., & Wagenmakers, E. J. (2012). A default Bayesian hypothesis test for correlations and partial correlations. *Psychonomic bulletin & review*, 19(6), 1057-1064.

Woolrich, M. W., Jbabdi, S., Patenaude, B., Chappell, M., Makni, S., Behrens, T., ... & Smith, S. M. (2009). Bayesian analysis of neuroimaging data in FSL. *Neuroimage*, 45(1), S173-S186.
